# Suppression and azimuthal anisotropy of prompt and nonprompt $${\mathrm{J}}/\psi $$ production in PbPb collisions at $$\sqrt{{s_{_{\text {NN}}}}} =2.76$$$$\,\mathrm{TeV}$$

**DOI:** 10.1140/epjc/s10052-017-4781-1

**Published:** 2017-04-19

**Authors:** V. Khachatryan, A. M. Sirunyan, A. Tumasyan, W. Adam, E. Asilar, T. Bergauer, J. Brandstetter, E. Brondolin, M. Dragicevic, J. Erö, M. Flechl, M. Friedl, R. Frühwirth, V. M. Ghete, C. Hartl, N. Hörmann, J. Hrubec, M. Jeitler, A. König, I. Krätschmer, D. Liko, T. Matsushita, I. Mikulec, D. Rabady, N. Rad, B. Rahbaran, H. Rohringer, J. Schieck, J. Strauss, W. Waltenberger, C.-E. Wulz, O. Dvornikov, V. Makarenko, V. Zykunov, V. Mossolov, N. Shumeiko, J. Suarez Gonzalez, S. Alderweireldt, E. A. De Wolf, X. Janssen, J. Lauwers, M. Van De Klundert, H. Van Haevermaet, P. Van Mechelen, N. Van Remortel, A. Van Spilbeeck, S. Abu Zeid, F. Blekman, J. D’Hondt, N. Daci, I. De Bruyn, K. Deroover, S. Lowette, S. Moortgat, L. Moreels, A. Olbrechts, Q. Python, S. Tavernier, W. Van Doninck, P. Van Mulders, I. Van Parijs, H. Brun, B. Clerbaux, G. De Lentdecker, H. Delannoy, G. Fasanella, L. Favart, R. Goldouzian, A. Grebenyuk, G. Karapostoli, T. Lenzi, A. Léonard, J. Luetic, T. Maerschalk, A. Marinov, A. Randle-conde, T. Seva, C. Vander Velde, P. Vanlaer, D. Vannerom, R. Yonamine, F. Zenoni, F. Zhang, A. Cimmino, T. Cornelis, D. Dobur, A. Fagot, G. Garcia, M. Gul, I. Khvastunov, D. Poyraz, S. Salva, R. Schöfbeck, A. Sharma, M. Tytgat, W. Van Driessche, E. Yazgan, N. Zaganidis, H. Bakhshiansohi, C. Beluffi, O. Bondu, S. Brochet, G. Bruno, A. Caudron, S. De Visscher, C. Delaere, M. Delcourt, B. Francois, A. Giammanco, A. Jafari, P. Jez, M. Komm, G. Krintiras, V. Lemaitre, A. Magitteri, A. Mertens, M. Musich, C. Nuttens, K. Piotrzkowski, L. Quertenmont, M. Selvaggi, M. Vidal Marono, S. Wertz, N. Beliy, W. L. Aldá Júnior, F. L. Alves, G. A. Alves, L. Brito, C. Hensel, A. Moraes, M. E. Pol, P. Rebello Teles, E. Belchior Batista Das Chagas, W. Carvalho, J. Chinellato, A. Custódio, E. M. Da Costa, G. G. Da Silveira, D. De Jesus Damiao, C. De Oliveira Martins, S. Fonseca De Souza, L. M. Huertas Guativa, H. Malbouisson, D. Matos Figueiredo, C. Mora Herrera, L. Mundim, H. Nogima, W. L. Prado Da Silva, A. Santoro, A. Sznajder, E. J. Tonelli Manganote, A. Vilela Pereira, S. Ahuja, C. A. Bernardes, S. Dogra, T. R. Fernandez Perez Tomei, E. M. Gregores, P. G. Mercadante, C. S. Moon, S. F. Novaes, Sandra S. Padula, D. Romero Abad, J. C. Ruiz Vargas, A. Aleksandrov, R. Hadjiiska, P. Iaydjiev, M. Rodozov, S. Stoykova, G. Sultanov, M. Vutova, A. Dimitrov, I. Glushkov, L. Litov, B. Pavlov, P. Petkov, W. Fang, M. Ahmad, J. G. Bian, G. M. Chen, H. S. Chen, M. Chen, Y. Chen, T. Cheng, C. H. Jiang, D. Leggat, Z. Liu, F. Romeo, S. M. Shaheen, A. Spiezia, J. Tao, C. Wang, Z. Wang, H. Zhang, J. Zhao, Y. Ban, G. Chen, Q. Li, S. Liu, Y. Mao, S. J. Qian, D. Wang, Z. Xu, C. Avila, A. Cabrera, L. F. Chaparro Sierra, C. Florez, J. P. Gomez, C. F. González Hernández, J. D. Ruiz Alvarez, J. C. Sanabria, N. Godinovic, D. Lelas, I. Puljak, P. M. Ribeiro Cipriano, T. Sculac, Z. Antunovic, M. Kovac, V. Brigljevic, D. Ferencek, K. Kadija, S. Micanovic, L. Sudic, T. Susa, A. Attikis, G. Mavromanolakis, J. Mousa, C. Nicolaou, F. Ptochos, P. A. Razis, H. Rykaczewski, D. Tsiakkouri, M. Finger, M. Finger, E. Carrera Jarrin, A. Ellithi Kamel, M. A. Mahmoud, A. Radi, M. Kadastik, L. Perrini, M. Raidal, A. Tiko, C. Veelken, P. Eerola, J. Pekkanen, M. Voutilainen, J. Härkönen, T. Järvinen, V. Karimäki, R. Kinnunen, T. Lampén, K. Lassila-Perini, S. Lehti, T. Lindén, P. Luukka, J. Tuominiemi, E. Tuovinen, L. Wendland, J. Talvitie, T. Tuuva, M. Besancon, F. Couderc, M. Dejardin, D. Denegri, B. Fabbro, J. L. Faure, C. Favaro, F. Ferri, S. Ganjour, S. Ghosh, A. Givernaud, P. Gras, G. Hamel de Monchenault, P. Jarry, I. Kucher, E. Locci, M. Machet, J. Malcles, J. Rander, A. Rosowsky, M. Titov, A. Zghiche, A. Abdulsalam, I. Antropov, S. Baffioni, F. Beaudette, P. Busson, L. Cadamuro, E. Chapon, C. Charlot, O. Davignon, R. Granier de Cassagnac, M. Jo, S. Lisniak, P. Miné, M. Nguyen, C. Ochando, G. Ortona, P. Paganini, P. Pigard, S. Regnard, R. Salerno, Y. Sirois, T. Strebler, Y. Yilmaz, A. Zabi, J.-L. Agram, J. Andrea, A. Aubin, D. Bloch, J.-M. Brom, M. Buttignol, E. C. Chabert, N. Chanon, C. Collard, E. Conte, X. Coubez, J.-C. Fontaine, D. Gelé, U. Goerlach, A.-C. Le Bihan, K. Skovpen, P. Van Hove, S. Gadrat, S. Beauceron, C. Bernet, G. Boudoul, E. Bouvier, C. A. Carrillo Montoya, R. Chierici, D. Contardo, B. Courbon, P. Depasse, H. El Mamouni, J. Fan, J. Fay, S. Gascon, M. Gouzevitch, G. Grenier, B. Ille, F. Lagarde, I. B. Laktineh, M. Lethuillier, L. Mirabito, A. L. Pequegnot, S. Perries, A. Popov, D. Sabes, V. Sordini, M. Vander Donckt, P. Verdier, S. Viret, T. Toriashvili, D. Lomidze, C. Autermann, S. Beranek, L. Feld, A. Heister, M. K. Kiesel, K. Klein, M. Lipinski, A. Ostapchuk, M. Preuten, F. Raupach, S. Schael, C. Schomakers, J. Schulz, T. Verlage, H. Weber, V. Zhukov, A. Albert, M. Brodski, E. Dietz-Laursonn, D. Duchardt, M. Endres, M. Erdmann, S. Erdweg, T. Esch, R. Fischer, A. Güth, M. Hamer, T. Hebbeker, C. Heidemann, K. Hoepfner, S. Knutzen, M. Merschmeyer, A. Meyer, P. Millet, S. Mukherjee, M. Olschewski, K. Padeken, T. Pook, M. Radziej, H. Reithler, M. Rieger, F. Scheuch, L. Sonnenschein, D. Teyssier, S. Thüer, V. Cherepanov, G. Flügge, B. Kargoll, T. Kress, A. Künsken, J. Lingemann, T. Müller, A. Nehrkorn, A. Nowack, C. Pistone, O. Pooth, A. Stahl, M. Aldaya Martin, T. Arndt, C. Asawatangtrakuldee, K. Beernaert, O. Behnke, U. Behrens, A. A. Bin Anuar, K. Borras, A. Campbell, P. Connor, C. Contreras-Campana, F. Costanza, C. Diez Pardos, G. Dolinska, G. Eckerlin, D. Eckstein, T. Eichhorn, E. Eren, E. Gallo, J. Garay Garcia, A. Geiser, A. Gizhko, J. M. Grados Luyando, P. Gunnellini, A. Harb, J. Hauk, M. Hempel, H. Jung, A. Kalogeropoulos, O. Karacheban, M. Kasemann, J. Keaveney, C. Kleinwort, I. Korol, D. Krücker, W. Lange, A. Lelek, J. Leonard, K. Lipka, A. Lobanov, W. Lohmann, R. Mankel, I.-A. Melzer-Pellmann, A. B. Meyer, G. Mittag, J. Mnich, A. Mussgiller, E. Ntomari, D. Pitzl, R. Placakyte, A. Raspereza, B. Roland, M.Ö. Sahin, P. Saxena, T. Schoerner-Sadenius, C. Seitz, S. Spannagel, N. Stefaniuk, G. P. Van Onsem, R. Walsh, C. Wissing, V. Blobel, M. Centis Vignali, A. R. Draeger, T. Dreyer, E. Garutti, D. Gonzalez, J. Haller, M. Hoffmann, A. Junkes, R. Klanner, R. Kogler, N. Kovalchuk, T. Lapsien, T. Lenz, I. Marchesini, D. Marconi, M. Meyer, M. Niedziela, D. Nowatschin, F. Pantaleo, T. Peiffer, A. Perieanu, J. Poehlsen, C. Sander, C. Scharf, P. Schleper, A. Schmidt, S. Schumann, J. Schwandt, H. Stadie, G. Steinbrück, F. M. Stober, M. Stöver, H. Tholen, D. Troendle, E. Usai, L. Vanelderen, A. Vanhoefer, B. Vormwald, M. Akbiyik, C. Barth, S. Baur, C. Baus, J. Berger, E. Butz, R. Caspart, T. Chwalek, F. Colombo, W. De Boer, A. Dierlamm, S. Fink, B. Freund, R. Friese, M. Giffels, A. Gilbert, P. Goldenzweig, D. Haitz, F. Hartmann, S. M. Heindl, U. Husemann, I. Katkov, S. Kudella, P. Lobelle Pardo, H. Mildner, M. U. Mozer, Th. Müller, M. Plagge, G. Quast, K. Rabbertz, S. Röcker, F. Roscher, M. Schröder, I. Shvetsov, G. Sieber, H. J. Simonis, R. Ulrich, J. Wagner-Kuhr, S. Wayand, M. Weber, T. Weiler, S. Williamson, C. Wöhrmann, R. Wolf, G. Anagnostou, G. Daskalakis, T. Geralis, V. A. Giakoumopoulou, A. Kyriakis, D. Loukas, I. Topsis-Giotis, S. Kesisoglou, A. Panagiotou, N. Saoulidou, E. Tziaferi, I. Evangelou, G. Flouris, C. Foudas, P. Kokkas, N. Loukas, N. Manthos, I. Papadopoulos, E. Paradas, N. Filipovic, G. Bencze, C. Hajdu, D. Horvath, F. Sikler, V. Veszpremi, G. Vesztergombi, A. J. Zsigmond, N. Beni, S. Czellar, J. Karancsi, A. Makovec, J. Molnar, Z. Szillasi, M. Bartók, P. Raics, Z. L. Trocsanyi, B. Ujvari, S. Bahinipati, S. Choudhury, P. Mal, K. Mandal, A. Nayak, D. K. Sahoo, N. Sahoo, S. K. Swain, S. Bansal, S. B. Beri, V. Bhatnagar, R. Chawla, U. Bhawandeep, A. K. Kalsi, A. Kaur, M. Kaur, R. Kumar, P. Kumari, A. Mehta, M. Mittal, J. B. Singh, G. Walia, Ashok Kumar, A. Bhardwaj, B. C. Choudhary, R. B. Garg, S. Keshri, S. Malhotra, M. Naimuddin, N. Nishu, K. Ranjan, R. Sharma, V. Sharma, R. Bhattacharya, S. Bhattacharya, K. Chatterjee, S. Dey, S. Dutt, S. Dutta, S. Ghosh, N. Majumdar, A. Modak, K. Mondal, S. Mukhopadhyay, S. Nandan, A. Purohit, A. Roy, D. Roy, S. Roy Chowdhury, S. Sarkar, M. Sharan, S. Thakur, P. K. Behera, R. Chudasama, D. Dutta, V. Jha, V. Kumar, A. K. Mohanty, P. K. Netrakanti, L. M. Pant, P. Shukla, A. Topkar, T. Aziz, S. Dugad, G. Kole, B. Mahakud, S. Mitra, G. B. Mohanty, B. Parida, N. Sur, B. Sutar, S. Banerjee, S. Bhowmik, R. K. Dewanjee, S. Ganguly, M. Guchait, Sa. Jain, S. Kumar, M. Maity, G. Majumder, K. Mazumdar, T. Sarkar, N. Wickramage, S. Chauhan, S. Dube, V. Hegde, A. Kapoor, K. Kothekar, S. Pandey, A. Rane, S. Sharma, H. Behnamian, S. Chenarani, E. Eskandari Tadavani, S. M. Etesami, A. Fahim, M. Khakzad, M. Mohammadi Najafabadi, M. Naseri, S. Paktinat Mehdiabadi, F. Rezaei Hosseinabadi, B. Safarzadeh, M. Zeinali, M. Felcini, M. Grunewald, M. Abbrescia, C. Calabria, C. Caputo, A. Colaleo, D. Creanza, L. Cristella, N. De Filippis, M. De Palma, L. Fiore, G. Iaselli, G. Maggi, M. Maggi, G. Miniello, S. My, S. Nuzzo, A. Pompili, G. Pugliese, R. Radogna, A. Ranieri, G. Selvaggi, L. Silvestris, R. Venditti, P. Verwilligen, G. Abbiendi, C. Battilana, D. Bonacorsi, S. Braibant-Giacomelli, L. Brigliadori, R. Campanini, P. Capiluppi, A. Castro, F. R. Cavallo, S. S. Chhibra, G. Codispoti, M. Cuffiani, G. M. Dallavalle, F. Fabbri, A. Fanfani, D. Fasanella, P. Giacomelli, C. Grandi, L. Guiducci, S. Marcellini, G. Masetti, A. Montanari, F. L. Navarria, A. Perrotta, A. M. Rossi, T. Rovelli, G. P. Siroli, N. Tosi, S. Albergo, S. Costa, A. Di Mattia, F. Giordano, R. Potenza, A. Tricomi, C. Tuve, G. Barbagli, V. Ciulli, C. Civinini, R. D’Alessandro, E. Focardi, P. Lenzi, M. Meschini, S. Paoletti, G. Sguazzoni, L. Viliani, L. Benussi, S. Bianco, F. Fabbri, D. Piccolo, F. Primavera, V. Calvelli, F. Ferro, M. Lo Vetere, M. R. Monge, E. Robutti, S. Tosi, L. Brianza, M. E. Dinardo, S. Fiorendi, S. Gennai, A. Ghezzi, P. Govoni, M. Malberti, S. Malvezzi, R. A. Manzoni, D. Menasce, L. Moroni, M. Paganoni, D. Pedrini, S. Pigazzini, S. Ragazzi, T. Tabarelli de Fatis, S. Buontempo, N. Cavallo, G. De Nardo, S. Di Guida, M. Esposito, F. Fabozzi, F. Fienga, A. O. M. Iorio, G. Lanza, L. Lista, S. Meola, P. Paolucci, C. Sciacca, F. Thyssen, P. Azzi, N. Bacchetta, L. Benato, D. Bisello, A. Boletti, R. Carlin, A. Carvalho Antunes De Oliveira, P. Checchia, M. Dall’Osso, P. De Castro Manzano, T. Dorigo, U. Dosselli, F. Gasparini, U. Gasparini, A. Gozzelino, S. Lacaprara, M. Margoni, A. T. Meneguzzo, J. Pazzini, N. Pozzobon, P. Ronchese, F. Simonetto, E. Torassa, M. Zanetti, P. Zotto, G. Zumerle, A. Braghieri, A. Magnani, P. Montagna, S. P. Ratti, V. Re, C. Riccardi, P. Salvini, I. Vai, P. Vitulo, L. Alunni Solestizi, G. M. Bilei, D. Ciangottini, L. Fanò, P. Lariccia, R. Leonardi, G. Mantovani, M. Menichelli, A. Saha, A. Santocchia, K. Androsov, P. Azzurri, G. Bagliesi, J. Bernardini, T. Boccali, R. Castaldi, M. A. Ciocci, R. Dell’Orso, S. Donato, G. Fedi, A. Giassi, M. T. Grippo, F. Ligabue, T. Lomtadze, L. Martini, A. Messineo, F. Palla, A. Rizzi, A. Savoy-Navarro, P. Spagnolo, R. Tenchini, G. Tonelli, A. Venturi, P. G. Verdini, L. Barone, F. Cavallari, M. Cipriani, D. Del Re, M. Diemoz, S. Gelli, E. Longo, F. Margaroli, B. Marzocchi, P. Meridiani, G. Organtini, R. Paramatti, F. Preiato, S. Rahatlou, C. Rovelli, F. Santanastasio, N. Amapane, R. Arcidiacono, S. Argiro, M. Arneodo, N. Bartosik, R. Bellan, C. Biino, N. Cartiglia, F. Cenna, M. Costa, R. Covarelli, A. Degano, N. Demaria, L. Finco, B. Kiani, C. Mariotti, S. Maselli, E. Migliore, V. Monaco, E. Monteil, M. Monteno, M. M. Obertino, L. Pacher, N. Pastrone, M. Pelliccioni, G. L. Pinna Angioni, F. Ravera, A. Romero, M. Ruspa, R. Sacchi, K. Shchelina, V. Sola, A. Solano, A. Staiano, P. Traczyk, S. Belforte, M. Casarsa, F. Cossutti, G. Della Ricca, A. Zanetti, D. H. Kim, G. N. Kim, M. S. Kim, S. Lee, S. W. Lee, Y. D. Oh, S. Sekmen, D. C. Son, Y. C. Yang, A. Lee, H. Kim, D. H. Moon, J. A. Brochero Cifuentes, T. J. Kim, S. Cho, S. Choi, Y. Go, D. Gyun, S. Ha, B. Hong, Y. Jo, Y. Kim, B. Lee, K. Lee, K. S. Lee, S. Lee, J. Lim, S. K. Park, Y. Roh, J. Almond, J. Kim, H. Lee, S. B. Oh, B. C. Radburn-Smith, S. H. Seo, U. K. Yang, H. D. Yoo, G. B. Yu, M. Choi, H. Kim, J. H. Kim, J. S. H. Lee, I. C. Park, G. Ryu, M. S. Ryu, Y. Choi, J. Goh, C. Hwang, J. Lee, I. Yu, V. Dudenas, A. Juodagalvis, J. Vaitkus, I. Ahmed, Z. A. Ibrahim, J. R. Komaragiri, M. A. B. Md Ali, F. Mohamad Idris, W. A. T. Wan Abdullah, M. N. Yusli, Z. Zolkapli, H. Castilla-Valdez, E. De La Cruz-Burelo, I. Heredia-De La Cruz, A. Hernandez-Almada, R. Lopez-Fernandez, R. Magaña Villalba, J. Mejia Guisao, A. Sanchez-Hernandez, S. Carrillo Moreno, C. Oropeza Barrera, F. Vazquez Valencia, S. Carpinteyro, I. Pedraza, H. A. Salazar Ibarguen, C. Uribe Estrada, A. Morelos Pineda, D. Krofcheck, P. H. Butler, A. Ahmad, M. Ahmad, Q. Hassan, H. R. Hoorani, W. A. Khan, A. Saddique, M. A. Shah, M. Shoaib, M. Waqas, H. Bialkowska, M. Bluj, B. Boimska, T. Frueboes, M. Górski, M. Kazana, K. Nawrocki, K. Romanowska-Rybinska, M. Szleper, P. Zalewski, K. Bunkowski, A. Byszuk, K. Doroba, A. Kalinowski, M. Konecki, J. Krolikowski, M. Misiura, M. Olszewski, M. Walczak, P. Bargassa, C. Beirão Da Cruz E Silva, B. Calpas, A. Di Francesco, P. Faccioli, P. G. Ferreira Parracho, M. Gallinaro, J. Hollar, N. Leonardo, L. Lloret Iglesias, M. V. Nemallapudi, J. Rodrigues Antunes, J. Seixas, O. Toldaiev, D. Vadruccio, J. Varela, P. Vischia, S. Afanasiev, P. Bunin, M. Gavrilenko, I. Golutvin, I. Gorbunov, A. Kamenev, V. Karjavin, A. Lanev, A. Malakhov, V. Matveev, V. Palichik, V. Perelygin, S. Shmatov, S. Shulha, N. Skatchkov, V. Smirnov, N. Voytishin, A. Zarubin, L. Chtchipounov, V. Golovtsov, Y. Ivanov, V. Kim, E. Kuznetsova, V. Murzin, V. Oreshkin, V. Sulimov, A. Vorobyev, Yu. Andreev, A. Dermenev, S. Gninenko, N. Golubev, A. Karneyeu, M. Kirsanov, N. Krasnikov, A. Pashenkov, D. Tlisov, A. Toropin, V. Epshteyn, V. Gavrilov, N. Lychkovskaya, V. Popov, I. Pozdnyakov, G. Safronov, A. Spiridonov, M. Toms, E. Vlasov, A. Zhokin, A. Bylinkin, R. Chistov, S. Polikarpov, V. Rusinov, V. Andreev, M. Azarkin, I. Dremin, M. Kirakosyan, A. Leonidov, A. Terkulov, A. Baskakov, A. Belyaev, E. Boos, A. Demiyanov, A. Ershov, A. Gribushin, O. Kodolova, V. Korotkikh, I. Lokhtin, I. Miagkov, S. Obraztsov, S. Petrushanko, V. Savrin, A. Snigirev, I. Vardanyan, V. Blinov, Y. Skovpen, D. Shtol, I. Azhgirey, I. Bayshev, S. Bitioukov, D. Elumakhov, V. Kachanov, A. Kalinin, D. Konstantinov, V. Krychkine, V. Petrov, R. Ryutin, A. Sobol, S. Troshin, N. Tyurin, A. Uzunian, A. Volkov, P. Adzic, P. Cirkovic, D. Devetak, M. Dordevic, J. Milosevic, V. Rekovic, J. Alcaraz Maestre, M. Barrio Luna, E. Calvo, M. Cerrada, M. Chamizo Llatas, N. Colino, B. De La Cruz, A. Delgado Peris, A. Escalante Del Valle, C. Fernandez Bedoya, J. P. Fernández Ramos, J. Flix, M. C. Fouz, P. Garcia-Abia, O. Gonzalez Lopez, S. Goy Lopez, J. M. Hernandez, M. I. Josa, E. Navarro De Martino, A. Pérez-Calero Yzquierdo, J. Puerta Pelayo, A. Quintario Olmeda, I. Redondo, L. Romero, M. S. Soares, J. F. de Trocóniz, M. Missiroli, D. Moran, J. Cuevas, J. Fernandez Menendez, I. Gonzalez Caballero, J. R. González Fernández, E. Palencia Cortezon, S. Sanchez Cruz, I. Suárez Andrés, J. M. Vizan Garcia, I. J. Cabrillo, A. Calderon, J. R. Castiñeiras De Saa, E. Curras, M. Fernandez, J. Garcia-Ferrero, G. Gomez, A. Lopez Virto, J. Marco, C. Martinez Rivero, F. Matorras, J. Piedra Gomez, T. Rodrigo, A. Ruiz-Jimeno, L. Scodellaro, N. Trevisani, I. Vila, R. Vilar Cortabitarte, D. Abbaneo, E. Auffray, G. Auzinger, M. Bachtis, P. Baillon, A. H. Ball, D. Barney, P. Bloch, A. Bocci, A. Bonato, C. Botta, T. Camporesi, R. Castello, M. Cepeda, G. Cerminara, M. D’Alfonso, D. d’Enterria, A. Dabrowski, V. Daponte, A. David, M. De Gruttola, A. De Roeck, E. Di Marco, M. Dobson, B. Dorney, T. du Pree, D. Duggan, M. Dünser, N. Dupont, A. Elliott-Peisert, S. Fartoukh, G. Franzoni, J. Fulcher, W. Funk, D. Gigi, K. Gill, M. Girone, F. Glege, D. Gulhan, S. Gundacker, M. Guthoff, J. Hammer, P. Harris, J. Hegeman, V. Innocente, P. Janot, J. Kieseler, H. Kirschenmann, V. Knünz, A. Kornmayer, M. J. Kortelainen, K. Kousouris, M. Krammer, C. Lange, P. Lecoq, C. Lourenço, M. T. Lucchini, L. Malgeri, M. Mannelli, A. Martelli, F. Meijers, J. A. Merlin, S. Mersi, E. Meschi, P. Milenovic, F. Moortgat, S. Morovic, M. Mulders, H. Neugebauer, S. Orfanelli, L. Orsini, L. Pape, E. Perez, M. Peruzzi, A. Petrilli, G. Petrucciani, A. Pfeiffer, M. Pierini, A. Racz, T. Reis, G. Rolandi, M. Rovere, M. Ruan, H. Sakulin, J. B. Sauvan, C. Schäfer, C. Schwick, M. Seidel, A. Sharma, P. Silva, P. Sphicas, J. Steggemann, M. Stoye, Y. Takahashi, M. Tosi, D. Treille, A. Triossi, A. Tsirou, V. Veckalns, G. I. Veres, M. Verweij, N. Wardle, H. K. Wöhri, A. Zagozdzinska, W. D. Zeuner, W. Bertl, K. Deiters, W. Erdmann, R. Horisberger, Q. Ingram, H. C. Kaestli, D. Kotlinski, U. Langenegger, T. Rohe, F. Bachmair, L. Bäni, L. Bianchini, B. Casal, G. Dissertori, M. Dittmar, M. Donegà, C. Grab, C. Heidegger, D. Hits, J. Hoss, G. Kasieczka, P. Lecomte, W. Lustermann, B. Mangano, M. Marionneau, P. Martinez Ruiz del Arbol, M. Masciovecchio, M. T. Meinhard, D. Meister, F. Micheli, P. Musella, F. Nessi-Tedaldi, F. Pandolfi, J. Pata, F. Pauss, G. Perrin, L. Perrozzi, M. Quittnat, M. Rossini, M. Schönenberger, A. Starodumov, V. R. Tavolaro, K. Theofilatos, R. Wallny, T. K. Aarrestad, C. Amsler, L. Caminada, M. F. Canelli, A. De Cosa, C. Galloni, A. Hinzmann, T. Hreus, B. Kilminster, J. Ngadiuba, D. Pinna, G. Rauco, P. Robmann, D. Salerno, Y. Yang, A. Zucchetta, V. Candelise, T. H. Doan, Sh. Jain, R. Khurana, M. Konyushikhin, C. M. Kuo, W. Lin, Y. J. Lu, A. Pozdnyakov, S. S. Yu, Arun Kumar, P. Chang, Y. H. Chang, Y. W. Chang, Y. Chao, K. F. Chen, P. H. Chen, C. Dietz, F. Fiori, W.-S. Hou, Y. Hsiung, Y. F. Liu, R.-S. Lu, M. Miñano Moya, E. Paganis, A. Psallidas, J. F. Tsai, Y. M. Tzeng, B. Asavapibhop, G. Singh, N. Srimanobhas, N. Suwonjandee, A. Adiguzel, S. Cerci, S. Damarseckin, Z. S. Demiroglu, C. Dozen, I. Dumanoglu, S. Girgis, G. Gokbulut, Y. Guler, I. Hos, E. E. Kangal, O. Kara, A. Kayis Topaksu, U. Kiminsu, M. Oglakci, G. Onengut, K. Ozdemir, D. Sunar Cerci, B. Tali, S. Turkcapar, I. S. Zorbakir, C. Zorbilmez, B. Bilin, S. Bilmis, B. Isildak, G. Karapinar, M. Yalvac, M. Zeyrek, E. Gülmez, M. Kaya, O. Kaya, E. A. Yetkin, T. Yetkin, A. Cakir, K. Cankocak, S. Sen, B. Grynyov, L. Levchuk, P. Sorokin, R. Aggleton, F. Ball, L. Beck, J. J. Brooke, D. Burns, E. Clement, D. Cussans, H. Flacher, J. Goldstein, M. Grimes, G. P. Heath, H. F. Heath, J. Jacob, L. Kreczko, C. Lucas, D. M. Newbold, S. Paramesvaran, A. Poll, T. Sakuma, S. Seif El Nasr-storey, D. Smith, V. J. Smith, A. Belyaev, C. Brew, R. M. Brown, L. Calligaris, D. Cieri, D. J. A. Cockerill, J. A. Coughlan, K. Harder, S. Harper, E. Olaiya, D. Petyt, C. H. Shepherd-Themistocleous, A. Thea, I. R. Tomalin, T. Williams, M. Baber, R. Bainbridge, O. Buchmuller, A. Bundock, D. Burton, S. Casasso, M. Citron, D. Colling, L. Corpe, P. Dauncey, G. Davies, A. De Wit, M. Della Negra, R. Di Maria, P. Dunne, A. Elwood, D. Futyan, Y. Haddad, G. Hall, G. Iles, T. James, R. Lane, C. Laner, R. Lucas, L. Lyons, A.-M. Magnan, S. Malik, L. Mastrolorenzo, J. Nash, A. Nikitenko, J. Pela, B. Penning, M. Pesaresi, D. M. Raymond, A. Richards, A. Rose, C. Seez, S. Summers, A. Tapper, K. Uchida, M. Vazquez Acosta, T. Virdee, J. Wright, S. C. Zenz, J. E. Cole, P. R. Hobson, A. Khan, P. Kyberd, D. Leslie, I. D. Reid, P. Symonds, L. Teodorescu, M. Turner, A. Borzou, K. Call, J. Dittmann, K. Hatakeyama, H. Liu, N. Pastika, S. I. Cooper, C. Henderson, P. Rumerio, C. West, D. Arcaro, A. Avetisyan, T. Bose, D. Gastler, D. Rankin, C. Richardson, J. Rohlf, L. Sulak, D. Zou, G. Benelli, E. Berry, D. Cutts, A. Garabedian, J. Hakala, U. Heintz, J. M. Hogan, O. Jesus, K. H. M. Kwok, E. Laird, G. Landsberg, Z. Mao, M. Narain, S. Piperov, S. Sagir, E. Spencer, R. Syarif, R. Breedon, G. Breto, D. Burns, M. Calderon De La Barca Sanchez, S. Chauhan, M. Chertok, J. Conway, R. Conway, P. T. Cox, R. Erbacher, C. Flores, G. Funk, M. Gardner, W. Ko, R. Lander, C. Mclean, M. Mulhearn, D. Pellett, J. Pilot, S. Shalhout, J. Smith, M. Squires, D. Stolp, M. Tripathi, C. Bravo, R. Cousins, A. Dasgupta, P. Everaerts, A. Florent, J. Hauser, M. Ignatenko, N. Mccoll, D. Saltzberg, C. Schnaible, E. Takasugi, V. Valuev, M. Weber, K. Burt, R. Clare, J. Ellison, J. W. Gary, S. M. A. Ghiasi Shirazi, G. Hanson, J. Heilman, P. Jandir, E. Kennedy, F. Lacroix, O. R. Long, M. Olmedo Negrete, M. I. Paneva, A. Shrinivas, W. Si, H. Wei, S. Wimpenny, B. R. Yates, J. G. Branson, G. B. Cerati, S. Cittolin, M. Derdzinski, A. Holzner, D. Klein, V. Krutelyov, J. Letts, I. Macneill, D. Olivito, S. Padhi, M. Pieri, M. Sani, V. Sharma, S. Simon, M. Tadel, A. Vartak, S. Wasserbaech, C. Welke, J. Wood, F. Würthwein, A. Yagil, G. Zevi Della Porta, N. Amin, R. Bhandari, J. Bradmiller-Feld, C. Campagnari, A. Dishaw, V. Dutta, M. Franco Sevilla, C. George, F. Golf, L. Gouskos, J. Gran, R. Heller, J. Incandela, S. D. Mullin, A. Ovcharova, H. Qu, J. Richman, D. Stuart, I. Suarez, J. Yoo, D. Anderson, A. Apresyan, J. Bendavid, A. Bornheim, J. Bunn, Y. Chen, J. Duarte, J. M. Lawhorn, A. Mott, H. B. Newman, C. Pena, M. Spiropulu, J. R. Vlimant, S. Xie, R. Y. Zhu, M. B. Andrews, V. Azzolini, T. Ferguson, M. Paulini, J. Russ, M. Sun, H. Vogel, I. Vorobiev, M. Weinberg, J. P. Cumalat, W. T. Ford, F. Jensen, A. Johnson, M. Krohn, T. Mulholland, K. Stenson, S. R. Wagner, J. Alexander, J. Chaves, J. Chu, S. Dittmer, K. Mcdermott, N. Mirman, G. Nicolas Kaufman, J. R. Patterson, A. Rinkevicius, A. Ryd, L. Skinnari, L. Soffi, S. M. Tan, Z. Tao, J. Thom, J. Tucker, P. Wittich, M. Zientek, D. Winn, S. Abdullin, M. Albrow, G. Apollinari, S. Banerjee, L. A. T. Bauerdick, A. Beretvas, J. Berryhill, P. C. Bhat, G. Bolla, K. Burkett, J. N. Butler, H. W. K. Cheung, F. Chlebana, S. Cihangir, M. Cremonesi, V. D. Elvira, I. Fisk, J. Freeman, E. Gottschalk, L. Gray, D. Green, S. Grünendahl, O. Gutsche, D. Hare, R. M. Harris, S. Hasegawa, J. Hirschauer, Z. Hu, B. Jayatilaka, S. Jindariani, M. Johnson, U. Joshi, B. Klima, B. Kreis, S. Lammel, J. Linacre, D. Lincoln, R. Lipton, T. Liu, R. Lopes De Sá, J. Lykken, K. Maeshima, N. Magini, J. M. Marraffino, S. Maruyama, D. Mason, P. McBride, P. Merkel, S. Mrenna, S. Nahn, C. Newman-Holmes, V. O’Dell, K. Pedro, O. Prokofyev, G. Rakness, L. Ristori, E. Sexton-Kennedy, A. Soha, W. J. Spalding, L. Spiegel, S. Stoynev, N. Strobbe, L. Taylor, S. Tkaczyk, N. V. Tran, L. Uplegger, E. W. Vaandering, C. Vernieri, M. Verzocchi, R. Vidal, M. Wang, H. A. Weber, A. Whitbeck, Y. Wu, D. Acosta, P. Avery, P. Bortignon, D. Bourilkov, A. Brinkerhoff, A. Carnes, M. Carver, D. Curry, S. Das, R. D. Field, I. K. Furic, J. Konigsberg, A. Korytov, J. F. Low, P. Ma, K. Matchev, H. Mei, G. Mitselmakher, D. Rank, L. Shchutska, D. Sperka, L. Thomas, J. Wang, S. Wang, J. Yelton, S. Linn, P. Markowitz, G. Martinez, J. L. Rodriguez, A. Ackert, J. R. Adams, T. Adams, A. Askew, S. Bein, B. Diamond, S. Hagopian, V. Hagopian, K. F. Johnson, A. Khatiwada, H. Prosper, A. Santra, R. Yohay, M. M. Baarmand, V. Bhopatkar, S. Colafranceschi, M. Hohlmann, D. Noonan, T. Roy, F. Yumiceva, M. R. Adams, L. Apanasevich, D. Berry, R. R. Betts, I. Bucinskaite, R. Cavanaugh, O. Evdokimov, L. Gauthier, C. E. Gerber, D. J. Hofman, K. Jung, P. Kurt, C. O’Brien, I. D. Sandoval Gonzalez, P. Turner, N. Varelas, H. Wang, Z. Wu, M. Zakaria, J. Zhang, B. Bilki, W. Clarida, K. Dilsiz, S. Durgut, R. P. Gandrajula, M. Haytmyradov, V. Khristenko, J.-P. Merlo, H. Mermerkaya, A. Mestvirishvili, A. Moeller, J. Nachtman, H. Ogul, Y. Onel, F. Ozok, A. Penzo, C. Snyder, E. Tiras, J. Wetzel, K. Yi, I. Anderson, B. Blumenfeld, A. Cocoros, N. Eminizer, D. Fehling, L. Feng, A. V. Gritsan, P. Maksimovic, C. Martin, M. Osherson, J. Roskes, U. Sarica, M. Swartz, M. Xiao, Y. Xin, C. You, A. Al-bataineh, P. Baringer, A. Bean, S. Boren, J. Bowen, C. Bruner, J. Castle, L. Forthomme, R. P. Kenny, S. Khalil, A. Kropivnitskaya, D. Majumder, W. Mcbrayer, M. Murray, S. Sanders, R. Stringer, J. D. Tapia Takaki, Q. Wang, A. Ivanov, K. Kaadze, Y. Maravin, A. Mohammadi, L. K. Saini, N. Skhirtladze, S. Toda, F. Rebassoo, D. Wright, C. Anelli, A. Baden, O. Baron, A. Belloni, B. Calvert, S. C. Eno, C. Ferraioli, J. A. Gomez, N. J. Hadley, S. Jabeen, R. G. Kellogg, T. Kolberg, J. Kunkle, Y. Lu, A. C. Mignerey, F. Ricci-Tam, Y. H. Shin, A. Skuja, M. B. Tonjes, S. C. Tonwar, D. Abercrombie, B. Allen, A. Apyan, R. Barbieri, A. Baty, R. Bi, K. Bierwagen, S. Brandt, W. Busza, I. A. Cali, Z. Demiragli, L. Di Matteo, G. Gomez Ceballos, M. Goncharov, D. Hsu, Y. Iiyama, G. M. Innocenti, M. Klute, D. Kovalskyi, K. Krajczar, Y. S. Lai, Y.-J. Lee, A. Levin, P. D. Luckey, B. Maier, A. C. Marini, C. Mcginn, C. Mironov, S. Narayanan, X. Niu, C. Paus, C. Roland, G. Roland, J. Salfeld-Nebgen, G. S. F. Stephans, K. Sumorok, K. Tatar, M. Varma, D. Velicanu, J. Veverka, J. Wang, T. W. Wang, B. Wyslouch, M. Yang, V. Zhukova, A. C. Benvenuti, R. M. Chatterjee, A. Evans, A. Finkel, A. Gude, P. Hansen, S. Kalafut, S. C. Kao, Y. Kubota, Z. Lesko, J. Mans, S. Nourbakhsh, N. Ruckstuhl, R. Rusack, N. Tambe, J. Turkewitz, J. G. Acosta, S. Oliveros, E. Avdeeva, R. Bartek, K. Bloom, D. R. Claes, A. Dominguez, C. Fangmeier, R. Gonzalez Suarez, R. Kamalieddin, I. Kravchenko, A. Malta Rodrigues, F. Meier, J. Monroy, J. E. Siado, G. R. Snow, B. Stieger, M. Alyari, J. Dolen, J. George, A. Godshalk, C. Harrington, I. Iashvili, J. Kaisen, A. Kharchilava, A. Kumar, A. Parker, S. Rappoccio, B. Roozbahani, G. Alverson, E. Barberis, A. Hortiangtham, A. Massironi, D. M. Morse, D. Nash, T. Orimoto, R. Teixeira De Lima, D. Trocino, R.-J. Wang, D. Wood, S. Bhattacharya, O. Charaf, K. A. Hahn, A. Kubik, A. Kumar, N. Mucia, N. Odell, B. Pollack, M. H. Schmitt, K. Sung, M. Trovato, M. Velasco, N. Dev, M. Hildreth, K. Hurtado Anampa, C. Jessop, D. J. Karmgard, N. Kellams, K. Lannon, N. Marinelli, F. Meng, C. Mueller, Y. Musienko, M. Planer, A. Reinsvold, R. Ruchti, G. Smith, S. Taroni, M. Wayne, M. Wolf, A. Woodard, J. Alimena, L. Antonelli, B. Bylsma, L. S. Durkin, S. Flowers, B. Francis, A. Hart, C. Hill, R. Hughes, W. Ji, B. Liu, W. Luo, D. Puigh, B. L. Winer, H. W. Wulsin, S. Cooperstein, O. Driga, P. Elmer, J. Hardenbrook, P. Hebda, D. Lange, J. Luo, D. Marlow, J. Mc Donald, T. Medvedeva, K. Mei, M. Mooney, J. Olsen, C. Palmer, P. Piroué, D. Stickland, A. Svyatkovskiy, C. Tully, A. Zuranski, S. Malik, A. Barker, V. E. Barnes, S. Folgueras, L. Gutay, M. K. Jha, M. Jones, A. W. Jung, D. H. Miller, N. Neumeister, J. F. Schulte, X. Shi, J. Sun, F. Wang, W. Xie, N. Parashar, J. Stupak, A. Adair, B. Akgun, Z. Chen, K. M. Ecklund, F. J. M. Geurts, M. Guilbaud, W. Li, B. Michlin, M. Northup, B. P. Padley, R. Redjimi, J. Roberts, J. Rorie, Z. Tu, J. Zabel, B. Betchart, A. Bodek, P. de Barbaro, R. Demina, Y. T. Duh, T. Ferbel, M. Galanti, A. Garcia-Bellido, J. Han, O. Hindrichs, A. Khukhunaishvili, K. H. Lo, P. Tan, M. Verzetti, A. Agapitos, J. P. Chou, E. Contreras-Campana, Y. Gershtein, T. A. Gómez Espinosa, E. Halkiadakis, M. Heindl, D. Hidas, E. Hughes, S. Kaplan, R. Kunnawalkam Elayavalli, S. Kyriacou, A. Lath, K. Nash, H. Saka, S. Salur, S. Schnetzer, D. Sheffield, S. Somalwar, R. Stone, S. Thomas, P. Thomassen, M. Walker, A. G. Delannoy, M. Foerster, J. Heideman, G. Riley, K. Rose, S. Spanier, K. Thapa, O. Bouhali, A. Celik, M. Dalchenko, M. De Mattia, A. Delgado, S. Dildick, R. Eusebi, J. Gilmore, T. Huang, E. Juska, T. Kamon, R. Mueller, Y. Pakhotin, R. Patel, A. Perloff, L. Perniè, D. Rathjens, A. Rose, A. Safonov, A. Tatarinov, K. A. Ulmer, N. Akchurin, C. Cowden, J. Damgov, F. De Guio, C. Dragoiu, P. R. Dudero, J. Faulkner, E. Gurpinar, S. Kunori, K. Lamichhane, S. W. Lee, T. Libeiro, T. Peltola, S. Undleeb, I. Volobouev, Z. Wang, S. Greene, A. Gurrola, R. Janjam, W. Johns, C. Maguire, A. Melo, H. Ni, P. Sheldon, S. Tuo, J. Velkovska, Q. Xu, M. W. Arenton, P. Barria, B. Cox, J. Goodell, R. Hirosky, A. Ledovskoy, H. Li, C. Neu, T. Sinthuprasith, X. Sun, Y. Wang, E. Wolfe, F. Xia, C. Clarke, R. Harr, P. E. Karchin, J. Sturdy, D. A. Belknap, J. Buchanan, C. Caillol, S. Dasu, L. Dodd, S. Duric, B. Gomber, M. Grothe, M. Herndon, A. Hervé, P. Klabbers, A. Lanaro, A. Levine, K. Long, R. Loveless, I. Ojalvo, T. Perry, G. A. Pierro, G. Polese, T. Ruggles, A. Savin, N. Smith, W. H. Smith, D. Taylor, N. Woods, CMS Collaboration

**Affiliations:** 1 Yerevan Physics Institute, Yerevan, Armenia; 2 Institut für Hochenergiephysik, Wien, Austria; 3 Institute for Nuclear Problems, Minsk, Belarus; 4 National Centre for Particle and High Energy Physics, Minsk, Belarus; 5 Universiteit Antwerpen, Antwerpen, Belgium; 6 Vrije Universiteit Brussel, Brussel, Belgium; 7 Université Libre de Bruxelles, Bruxelles, Belgium; 8 Ghent University, Ghent, Belgium; 9 Université Catholique de Louvain, Louvain-la-Neuve, Belgium; 10 Université de Mons, Mons, Belgium; 11 Centro Brasileiro de Pesquisas Fisicas, Rio de Janeiro, Brazil; 12 Universidade do Estado do Rio de Janeiro, Rio de Janeiro, Brazil; 13 Universidade Estadual Paulista, Universidade Federal do ABC, São Paulo, Brazil; 14 Institute for Nuclear Research and Nuclear Energy, Sofia, Bulgaria; 15 University of Sofia, Sofia, Bulgaria; 16 Beihang University, Beijing, China; 17 Institute of High Energy Physics, Beijing, China; 18 State Key Laboratory of Nuclear Physics and Technology, Peking University, Beijing, China; 19 Universidad de Los Andes, Bogota, Colombia; 20 University of Split, Faculty of Electrical Engineering, Mechanical Engineering and Naval Architecture, Split, Croatia; 21 University of Split, Faculty of Science, Split, Croatia; 22 Institute Rudjer Boskovic, Zagreb, Croatia; 23 University of Cyprus, Nicosia, Cyprus; 24 Charles University, Prague, Czech Republic; 25 Universidad San Francisco de Quito, Quito, Ecuador; 26 Academy of Scientific Research and Technology of the Arab Republic of Egypt, Egyptian Network of High Energy Physics, Cairo, Egypt; 27 National Institute of Chemical Physics and Biophysics, Tallinn, Estonia; 28 Department of Physics, University of Helsinki, Helsinki, Finland; 29 Helsinki Institute of Physics, Helsinki, Finland; 30 Lappeenranta University of Technology, Lappeenranta, Finland; 31 IRFU, CEA, Université Paris-Saclay, Gif-sur-Yvette, France; 32 Laboratoire Leprince-Ringuet, Ecole Polytechnique, IN2P3-CNRS, Palaiseau, France; 33 Institut Pluridisciplinaire Hubert Curien, Université de Strasbourg, Université de Haute Alsace Mulhouse, CNRS/IN2P3, Strasbourg, France; 34 Centre de Calcul de l’Institut National de Physique Nucleaire et de Physique des Particules, CNRS/IN2P3, Villeurbanne, France; 35 Université de Lyon, Université Claude Bernard Lyon 1, CNRS-IN2P3, Institut de Physique Nucléaire de Lyon, Villeurbanne, France; 36 Georgian Technical University, Tbilisi, Georgia; 37 Tbilisi State University, Tbilisi, Georgia; 38 RWTH Aachen University, I. Physikalisches Institut, Aachen, Germany; 39 RWTH Aachen University, III. Physikalisches Institut A, Aachen, Germany; 40 RWTH Aachen University, III. Physikalisches Institut B, Aachen, Germany; 41 Deutsches Elektronen-Synchrotron, Hamburg, Germany; 42 University of Hamburg, Hamburg, Germany; 43 Institut für Experimentelle Kernphysik, Karlsruhe, Germany; 44 Institute of Nuclear and Particle Physics (INPP), NCSR Demokritos, Aghia Paraskevi, Greece; 45 National and Kapodistrian University of Athens, Athens, Greece; 46 University of Ioánnina, Ioannina, Greece; 47 MTA-ELTE Lendület CMS Particle and Nuclear Physics Group, Eötvös Loránd University, Budapest, Hungary; 48 Wigner Research Centre for Physics, Budapest, Hungary; 49 Institute of Nuclear Research ATOMKI, Debrecen, Hungary; 50 University of Debrecen, Debrecen, Hungary; 51 National Institute of Science Education and Research, Bhubaneswar, India; 52 Panjab University, Chandigarh, India; 53 University of Delhi, Delhi, India; 54 Saha Institute of Nuclear Physics, Kolkata, India; 55 Indian Institute of Technology Madras, Madras, India; 56 Bhabha Atomic Research Centre, Mumbai, India; 57 Tata Institute of Fundamental Research-A, Mumbai, India; 58 Tata Institute of Fundamental Research-B, Mumbai, India; 59 Indian Institute of Science Education and Research (IISER), Pune, India; 60 Institute for Research in Fundamental Sciences (IPM), Tehran, Iran; 61 University College Dublin, Dublin, Ireland; 62 INFN Sezione di Bari, Università di Bari, Politecnico di Bari, Bari, Italy; 63 INFN Sezione di Bologna, Università di Bologna, Bologna, Italy; 64 INFN Sezione di Catania, Università di Catania, Catania, Italy; 65 INFN Sezione di Firenze, Università di Firenze, Firenze, Italy; 66 INFN Laboratori Nazionali di Frascati, Frascati, Italy; 67 INFN Sezione di Genova, Università di Genova, Genova, Italy; 68 INFN Sezione di Milano-Bicocca, Università di Milano-Bicocca, Milano, Italy; 69 INFN Sezione di Napoli, Università di Napoli ’Federico II’, Napoli, Italy, Università della Basilicata, Potenza, Italy, Università G. Marconi, Rome, Italy; 70 INFN Sezione di Padova, Università di Padova, Padova, Italy, Università di Trento, Trento, Italy; 71 INFN Sezione di Pavia, Università di Pavia, Pavia, Italy; 72 INFN Sezione di Perugia, Università di Perugia, Perugia, Italy; 73 INFN Sezione di Pisa, Università di Pisa, Scuola Normale Superiore di Pisa, Pisa, Italy; 74 INFN Sezione di Roma, Università di Roma, Rome, Italy; 75 INFN Sezione di Torino, Università di Torino, Torino, Italy, Università del Piemonte Orientale, Novara, Italy; 76 INFN Sezione di Trieste, Università di Trieste, Trieste, Italy; 77 Kyungpook National University, Daegu, Korea; 78 Chonbuk National University, Jeonju, Korea; 79 Chonnam National University, Institute for Universe and Elementary Particles, Kwangju, Korea; 80 Hanyang University, Seoul, Korea; 81 Korea University, Seoul, Korea; 82 Seoul National University, Seoul, Korea; 83 University of Seoul, Seoul, Korea; 84 Sungkyunkwan University, Suwon, Korea; 85 Vilnius University, Vilnius, Lithuania; 86 National Centre for Particle Physics, Universiti Malaya, Kuala Lumpur, Malaysia; 87 Centro de Investigacion y de Estudios Avanzados del IPN, Mexico City, Mexico; 88 Universidad Iberoamericana, Mexico City, Mexico; 89 Benemerita Universidad Autonoma de Puebla, Puebla, Mexico; 90 Universidad Autónoma de San Luis Potosí, San Luis Potosí, Mexico; 91 University of Auckland, Auckland, New Zealand; 92 University of Canterbury, Christchurch, New Zealand; 93 National Centre for Physics, Quaid-I-Azam University, Islamabad, Pakistan; 94 National Centre for Nuclear Research, Swierk, Poland; 95 Institute of Experimental Physics, Faculty of Physics, University of Warsaw, Warsaw, Poland; 96 Laboratório de Instrumentação e Física Experimental de Partículas, Lisboa, Portugal; 97 Joint Institute for Nuclear Research, Dubna, Russia; 98 Petersburg Nuclear Physics Institute, Gatchina, St. Petersburg, Russia; 99 Institute for Nuclear Research, Moscow, Russia; 100 Institute for Theoretical and Experimental Physics, Moscow, Russia; 101 Moscow Institute of Physics and Technology, Dolgoprudny, Russia; 102 National Research Nuclear University ‘Moscow Engineering Physics Institute’ (MEPhI), Moscow, Russia; 103 P.N. Lebedev Physical Institute, Moscow, Russia; 104 Skobeltsyn Institute of Nuclear Physics, Lomonosov Moscow State University, Moscow, Russia; 105 Novosibirsk State University (NSU), Novosibirsk, Russia; 106 State Research Center of Russian Federation, Institute for High Energy Physics, Protvino, Russia; 107 University of Belgrade, Faculty of Physics and Vinca Institute of Nuclear Sciences, Belgrade, Serbia; 108 Centro de Investigaciones Energéticas Medioambientales y Tecnológicas (CIEMAT), Madrid, Spain; 109 Universidad Autónoma de Madrid, Madrid, Spain; 110 Universidad de Oviedo, Oviedo, Spain; 111 Instituto de Física de Cantabria (IFCA), CSIC-Universidad de Cantabria, Santander, Spain; 112 CERN, European Organization for Nuclear Research, Geneva, Switzerland; 113 Paul Scherrer Institut, Villigen, Switzerland; 114 Institute for Particle Physics, ETH Zurich, Zurich, Switzerland; 115 Universität Zürich, Zurich, Switzerland; 116 National Central University, Chung-Li, Taiwan; 117 National Taiwan University (NTU), Taipei, Taiwan; 118 Chulalongkorn University, Faculty of Science, Department of Physics, Bangkok, Thailand; 119 Cukurova University, Adana, Turkey; 120 Middle East Technical University, Physics Department, Ankara, Turkey; 121 Bogazici University, Istanbul, Turkey; 122 Istanbul Technical University, Istanbul, Turkey; 123 Institute for Scintillation Materials of National Academy of Science of Ukraine, Kharkov, Ukraine; 124 National Scientific Center, Kharkov Institute of Physics and Technology, Kharkov, Ukraine; 125 University of Bristol, Bristol, UK; 126 Rutherford Appleton Laboratory, Didcot, UK; 127 Imperial College, London, UK; 128 Brunel University, Uxbridge, UK; 129 Baylor University, Waco, USA; 130 The University of Alabama, Tuscaloosa, USA; 131 Boston University, Boston, USA; 132 Brown University, Providence, USA; 133 University of California, Davis, Davis USA; 134 University of California, Los Angeles, USA; 135 University of California, Riverside, Riverside USA; 136 University of California, San Diego, La Jolla USA; 137 Santa Barbara-Department of Physics, University of California, Santa Barbara, USA; 138 California Institute of Technology, Pasadena, USA; 139 Carnegie Mellon University, Pittsburgh, USA; 140 University of Colorado Boulder, Boulder, USA; 141 Cornell University, Ithaca, USA; 142 Fairfield University, Fairfield, USA; 143 Fermi National Accelerator Laboratory, Batavia, USA; 144 University of Florida, Gainesville, USA; 145 Florida International University, Miami, USA; 146 Florida State University, Tallahassee, USA; 147 Florida Institute of Technology, Melbourne, USA; 148 University of Illinois at Chicago (UIC), Chicago, USA; 149 The University of Iowa, Iowa City, USA; 150 Johns Hopkins University, Baltimore, USA; 151 The University of Kansas, Lawrence, USA; 152 Kansas State University, Manhattan, USA; 153 Lawrence Livermore National Laboratory, Livermore, USA; 154 University of Maryland, College Park, USA; 155 Massachusetts Institute of Technology, Cambridge, USA; 156 University of Minnesota, Minneapolis, USA; 157 University of Mississippi, Oxford, USA; 158 University of Nebraska-Lincoln, Lincoln, USA; 159 State University of New York at Buffalo, Buffalo, USA; 160 Northeastern University, Boston, USA; 161 Northwestern University, Evanston, USA; 162 University of Notre Dame, Notre Dame, USA; 163 The Ohio State University, Columbus, USA; 164 Princeton University, Princeton, USA; 165 University of Puerto Rico, Mayaguez, USA; 166 Purdue University, West Lafayette, USA; 167 Purdue University Calumet, Hammond, USA; 168 Rice University, Houston, USA; 169 University of Rochester, Rochester, USA; 170 Rutgers, The State University of New Jersey, Piscataway, USA; 171 University of Tennessee, Knoxville, USA; 172 Texas A&M University, College Station, USA; 173 Texas Tech University, Lubbock, USA; 174 Vanderbilt University, Nashville, USA; 175 University of Virginia, Charlottesville, USA; 176 Wayne State University, Detroit, USA; 177 University of Wisconsin-Madison, Madison, WI USA; 178 CERN, 1211 Geneva 23, Switzerland

## Abstract

The nuclear modification factor $R_{\mathrm{AA}}$ and the azimuthal anisotropy coefficient $v_{2}$ of prompt and nonprompt (i.e. those from decays of b hadrons) ${\mathrm{J}}/\psi$ mesons, measured from PbPb and pp collisions at $\sqrt{{s_{_{\text {NN}}}}} =2.76$ $\,\mathrm{TeV}$ at the LHC, are reported. The results are presented in several event centrality intervals and several kinematic regions, for transverse momenta $p_{\mathrm{T}} >6.5$ $\,{\mathrm{GeV}}/{\mathrm{c}}$ and rapidity $|{y}|<2.4$, extending down to $p_{\mathrm{T}} =3$ $\,{\mathrm{GeV}}/{\mathrm{c}}$ in the $1.6<|{y}|<2.4$ range. The $v_{2}$ of prompt ${\mathrm{J}}/\psi$ is found to be nonzero, but with no strong dependence on centrality, rapidity, or $p_{\mathrm{T}}$ over the full kinematic range studied. The measured $v_{2}$ of nonprompt ${\mathrm{J}}/\psi$ is consistent with zero. The $R_{\mathrm{AA}}$ of prompt ${\mathrm{J}}/\psi$ exhibits a suppression that increases from peripheral to central collisions but does not vary strongly as a function of either *y* or $p_{\mathrm{T}}$ in the fiducial range. The nonprompt ${\mathrm{J}}/\psi$ $R_{\mathrm{AA}}$ shows a suppression which becomes stronger as rapidity or $p_{\mathrm{T}}$ increases. The $v_{2}$ and $R_{\mathrm{AA}}$ of open and hidden charm, and of open charm and beauty, are compared.

## Introduction

Recent data from RHIC and the CERN LHC for mesons containing charm and beauty quarks have allowed more detailed theoretical and experimental studies [1] of the phenomenology of these heavy quarks in a deconfined quark gluon plasma (QGP) [2] at large energy densities and high temperatures [3]. Heavy quarks, whether as quarkonium states $\mathrm{Q} \overline{\mathrm{Q}}$ (hidden heavy flavour) [4] or as mesons made of heavy-light quark–antiquark pairs $\mathrm{Q} \overline{\mathrm{q}}$ (open heavy flavour) [5], are considered key probes of the QGP, since their short formation time allows them to probe all stages of the QGP evolution [1].

At LHC energies, the inclusive ${\mathrm{J}}/\psi$ yield contains a significant nonprompt contribution from $\mathrm{b}$ hadron decays [6–8], offering the opportunity of studying both open beauty and hidden charm in the same measurement. Because of the long lifetime ($\mathcal {O}(500)\,\upmu \mathrm{m}/c$) of $\mathrm{b}$ hadrons, compared to the QGP lifetime ($\mathcal {O}(10) {fm}/c$), the nonprompt contribution should not suffer from colour screening of the potential between the $\mathrm{Q}$ and the $\overline{\mathrm{Q}}$ by the surrounding light quarks and gluons, which decreases the prompt quarkonium yield [9]. Instead, the nonprompt contribution should reflect the energy loss of $\mathrm{b}$ quarks in the medium. The importance of an unambiguous and detailed measurement of open beauty flavour is driven by the need to understand key features of the dynamics of parton interactions and hadron formation in the QGP: the colour-charge and parton-mass dependences for the in-medium interactions [5, 10–13], the relative contribution of radiative and collisional energy loss [14–16], and the effects of different hadron formation times [17, 18]. Another aspect of the heavy-quark phenomenology in the QGP concerns differences in the behaviour (energy loss mechanisms, amount and strength of interactions with the surrounding medium) of a $\mathrm{Q} \overline{\mathrm{Q}}$ pair (the pre-quarkonium state) relative to that of a single heavy quark *Q* (the pre-meson component) [19–21].

Experimentally, modifications to the particle production are usually quantified by the ratio of the yield measured in heavy ion collisions to that in proton–proton ($\mathrm{p}\mathrm{p}$) collisions, scaled by the mean number of binary nucleon–nucleon (NN) collisions. This ratio is called the nuclear modification factor $R_{\mathrm{AA}}$. In the absence of medium effects, one would expect $R_{\mathrm{AA}} = 1$ for hard processes, which scale with the number of NN collisions. The $R_{\mathrm{AA}}$ for prompt and nonprompt ${\mathrm{J}}/\psi$ have been previously measured in $\mathrm{PbPb}$ at $\sqrt{{s_{_{\text {NN}}}}} =2.76$ $\,\mathrm{TeV}$ by CMS in bins of transverse momentum ($p_{\mathrm{T}}$), rapidity (*y*) and collision centrality [22]. A strong centrality-dependent suppression has been observed for ${\mathrm{J}}/\psi$ with $p_{\mathrm{T}} > 6.5\,{\mathrm{GeV}}/{\mathrm{c}}$. The ALICE Collaboration has measured ${\mathrm{J}}/\psi$ down to $p_{\mathrm{T}} =0$ $\,{\mathrm{GeV}}/{\mathrm{c}}$ in the electron channel at midrapidity ($|{y}|<0.8$) [23] and in the muon channel at forward rapidity ($2.5<y<4$) [24]. Except for the most peripheral event selection, a suppression of inclusive ${\mathrm{J}}/\psi$ meson production is observed for all collision centralities. However, the suppression is smaller than that at $\sqrt{{s_{_{\text {NN}}}}} =0.2$ $\,\mathrm{TeV}$  [25], smaller at midrapidity than at forward rapidity, and, in the forward region, smaller for $p_{\mathrm{T}} <2$ $\,{\mathrm{GeV}}/{\mathrm{c}}$ than for $5<p_{\mathrm{T}} <8$ $\,{\mathrm{GeV}}/{\mathrm{c}}$  [26]. All these results were interpreted as evidence that the measured prompt ${\mathrm{J}}/\psi$ yield is the result of an interplay between (a) primordial production (${\mathrm{J}}/\psi$ produced in the initial hard-scattering of the collisions), (b) colour screening and energy loss (${\mathrm{J}}/\psi$ destroyed or modified by interactions with the surrounding medium), and (c) recombination/regeneration mechanisms in a deconfined partonic medium, or at the time of hadronization (${\mathrm{J}}/\psi$ created when a free charm and a free anti-charm quark come close enough to each other to form a bound state) [27–29].

A complement to the $$R_{\mathrm{AA}}$$ measurement is the elliptic anisotropy coefficient $$v_{2}$$. This is the second Fourier coefficient in the expansion of the azimuthal angle ($$\Phi $$) distribution of the $${\mathrm{J}}/\psi $$ mesons, $$\mathrm{d}N/\mathrm{d}\Phi \propto 1+2v_{2} \cos [2(\Phi -\Psi _{\mathrm{PP}})]$$ with respect to $$\Psi _{\mathrm{PP}}$$, the azimuthal angle of the “participant plane” calculated for each event. In a noncentral heavy ion collision, the overlap region of the two colliding nuclei has a lenticular shape. The participant plane is defined by the beam direction and the direction of the shorter axis of the lenticular region. Typical sources for a nonzero elliptic anisotropy are a path length difference arising from energy loss of particles traversing the reaction zone, or different pressure gradients along the short and long axes. Both effects convert the initial spatial anisotropy into a momentum anisotropy $$v_{2} $$ [30]. The effect of energy loss is usually studied using high $$p_{\mathrm{T}}$$ and/or heavy particles (so-called “hard probes” of the medium), for which the parent parton is produced at an early stage of the collision. If the partons are emitted in the direction of the participant plane, they have on average a shorter in-medium path length than partons emitted orthogonally, leading to a smaller modification to their energy or, in the case of $$\mathrm{Q} \overline{\mathrm{Q}} $$ and the corresponding onium state, a smaller probability of being destroyed. Pressure gradients drive in-medium interactions that can modify the direction of the partons. This effect is most important at low $$p_{\mathrm{T}}$$.

The $v_{2}$ of open charm (D mesons) and hidden charm (inclusive ${\mathrm{J}}/\psi$ mesons) was measured at the LHC by the ALICE Collaboration. The D mesons with $2<p_{\mathrm{T}} <6$ $\,{\mathrm{GeV}}/{\mathrm{c}}$  [31] were found to have a significant positive $v_{2}$, while for ${\mathrm{J}}/\psi$ mesons with $2<p_{\mathrm{T}} <4$ $\,{\mathrm{GeV}}/{\mathrm{c}}$ there was an indication of nonzero $v_{2}$  [32]. The precision of the results does not yet allow a determination of the origin of the observed anisotropy. One possible interpretation is that charm quarks at low $p_{\mathrm{T}}$, despite their much larger mass than those of the *u*, *s*, *d* quarks, participate in the collective expansion of the medium. A second possibility is that there is no collective motion for the charm quarks, and the observed anisotropy is acquired via quark recombination [27, 33, 34].

In this paper, the $R_{\mathrm{AA}}$ and the $v_{2}$ for prompt and nonprompt ${\mathrm{J}}/\psi$ mesons are presented in several event centrality intervals and several kinematic regions. The results are based on event samples collected during the 2011 $\mathrm{PbPb}$ and 2013 $\mathrm{p}\mathrm{p}$ LHC runs at a nucleon–nucleon centre-of-mass energy of 2.76$\,\mathrm{TeV}$, corresponding to integrated luminosities of 152$\,\upmu \mathrm{b}^{-1}$ and 5.4$\,{\mathrm{pb}}^{-1}$, respectively.

## Experimental setup and event selection

A detailed description of the CMS detector, together with a definition of the coordinate system and the relevant kinematic variables, can be found in Ref. [35]. The central feature of the CMS apparatus is a superconducting solenoid, of 6 m internal diameter and 15 m length. Within the field volume are the silicon tracker, the crystal electromagnetic calorimeter, and the brass and scintillator hadron calorimeter. The CMS apparatus also has extensive forward calorimetry, including two steel and quartz-fiber Cherenkov hadron forward (HF) calorimeters, which cover the range $2.9<|{\eta _\text {det}}|<5.2$, where $\eta _\text {det}$ is measured from the geometrical centre of the CMS detector. The calorimeter cells, in the $\eta$-$\phi$ plane, form towers projecting radially outwards from close to the nominal interaction point. These detectors are used in the present analysis for the event selection, collision impact parameter determination, and measurement of the azimuthal angle of the participant plane.

Muons are detected in the pseudorapidity window $|{\eta }|< 2.4$, by gas-ionization detectors made of three technologies: drift tubes, cathode strip chambers, and resistive plate chambers, embedded in the steel flux-return yoke of the solenoid. The silicon tracker is composed of pixel detectors (three barrel layers and two forward disks on either side of the detector, made of 66 million $100\times 150\,\upmu \mathrm{m} ^2$ pixels) followed by microstrip detectors (ten barrel layers plus three inner disks and nine forward disks on either side of the detector, with strip pitch between 80 and 180$\,\upmu \mathrm{m}$).

The measurements reported here are based on $\mathrm{PbPb}$ and $\mathrm{p}\mathrm{p}$ events selected online (triggered) by a hardware-based dimuon trigger without an explicit muon momentum threshold (i.e. the actual threshold is determined by the detector acceptance and efficiency of the muon trigger). The same trigger logic was used during the pp and PbPb data taking periods.

In order to select a sample of purely inelastic hadronic PbPb (pp) collisions, the contributions from ultraperipheral collisions and noncollision beam background are removed offline, as described in Ref. [36]. Events are preselected if they contain a reconstructed primary vertex formed by at least two tracks and at least three (one in the case of pp events) HF towers on each side of the interaction point with an energy of at least 3$\,\mathrm{GeV}$ deposited in each tower. To further suppress the beam-gas events, the distribution of hits in the pixel detector along the beam direction is required to be compatible with particles originating from the event vertex. These criteria select $(97\pm 3)$% (>99%) of inelastic hadronic PbPb (pp) collisions with negligible contamination from non-hadronic interactions [36]. Using this efficiency it is calculated that the $\mathrm{PbPb}$ sample corresponds to a number of minimum bias (MB) events $N_{\mathrm{MB}}=(1.16\pm 0.04)\times 10^9$. The pp data set corresponds to an integrated luminosity of 5.4$\,{\mathrm{pb}}^{-1}$ known to an accuracy of 3.7% from the uncertainty in the calibration based on a van der Meer scan [37]. The two data sets correspond to approximately the same number of elementary NN collisions.

Muons are reconstructed offline using tracks in the muon detectors (“standalone muons”) that are then matched to tracks in the silicon tracker, using an algorithm optimized for the heavy ion environment [38]. In addition, an iterative track reconstruction algorithm [39] is applied to the $\mathrm{PbPb}$ data, limited to regions defined by the standalone muons. The $\mathrm{p}\mathrm{p}$ reconstruction algorithm includes an iterative tracking step in the full silicon tracker. The final parameters of the muon trajectory are obtained from a global fit of the standalone muon with a matching track in the silicon tracker.

The centrality of heavy ion collisions, i.e. the geometrical overlap of the incoming nuclei, is correlated to the energy released in the collisions. In CMS, centrality is defined as percentiles of the distribution of the energy deposited in the HFs. Using a Glauber model calculation as described in Ref. [36], one can estimate variables related to the centrality, such as the mean number of nucleons participating in the collisions ($N_{\text {part}}$), the mean number of binary NN collisions ($N_{\text {coll}}$), and the average nuclear overlap function ($T_{\mathrm{AA}}$) [40]. The latter is equal to the number of NN binary collisions divided by the NN cross section and can be interpreted as the NN-equivalent integrated luminosity per heavy ion collision, at a given centrality. In the following, $N_{\text {part}}$ will be the variable used to show the centrality dependence of the measurements, while $T_{\mathrm{AA}}$ directly enters into the nuclear modification factor calculation. It should be noted that the PbPb hadronic cross section ($7.65 \pm 0.42$ b), computed with this Glauber simulation, results in an integrated luminosity of $152\pm 9$ $\,\upmu \mathrm{b}^{-1}$, compatible within 1.2 sigma with the integrated luminosity based on the van der Meer scan, which has been evaluated to be $166\pm 8$ $\,\upmu \mathrm{b}^{-1}$. All the $R_{\mathrm{AA}}$ results presented in the paper have been obtained using the $N_{\mathrm{MB}}$ event counting that is equivalent to 152$\,\upmu \mathrm{b}^{-1}$ expressed in terms of integrated luminosity.

Several Monte Carlo (MC) simulated event samples are used to model the signal shapes and evaluate reconstruction, trigger, and selection efficiencies. Samples of prompt and nonprompt ${\mathrm{J}}/\psi$ are generated with pythia  6.424 [41] and decayed with evtgen 1.3.0 [42], while the final-state bremsstrahlung is simulated with photos 2.0 [43]. The prompt ${\mathrm{J}}/\psi$ is simulated unpolarized, a scenario in good agreement with $\mathrm{p}\mathrm{p}$ measurements [44–46]. For nonprompt ${\mathrm{J}}/\psi$, the results are reported for the polarization predicted by evtgen, roughly $\lambda _{\theta } = -0.4$, however not a well-defined value, since in many ${\mathrm{B}}\rightarrow {\mathrm{J}}/\psi X$ modes the spin alignment is either forced by angular momentum conservation or given as input from measured values of helicity amplitudes in decays. If the acceptances were different in $\mathrm{p}\mathrm{p}$ and $\mathrm{PbPb}$, they would not perfectly cancel in the $R_{\mathrm{AA}}$. This would be the case if, for instance, some physics processes (such as polarization or energy loss) would affect the measurement in $\mathrm{PbPb}$ collisions with a strong kinematic dependence within an analysis bin. As in previous analyses [47–50], such possible physics effects are not considered as systematic uncertainties, but a quantitative estimate of this effect for two extreme polarization scenarios can be found in Ref. [22]. In the $\mathrm{PbPb}$ case, the pythia signal events are further embedded in heavy ion events generated with hydjet  1.8 [51], at the level of detector hits and with matching vertices. The detector response was simulated with Geant4  [52], and the resulting information was processed through the full event reconstruction chain, including trigger emulation.

## Analysis

Throughout this analysis the same methods for signal extraction and corrections are used for both the $\mathrm{p}\mathrm{p}$ and $\mathrm{PbPb}$ data.

### Corrections

For both $R_{\mathrm{AA}}$ and $v_{2}$ results, correction factors are applied event-by-event to each dimuon, to account for inefficiencies in the trigger, reconstruction, and selection of the $\mu ^{+} \mu ^{-}$ pairs. They were evaluated, using MC samples, in four dimensions ($p_{\mathrm{T}}$, centrality, *y*, and $L_{xyz}$) for the $\mathrm{PbPb}$ results, and in three-dimensions ($p_{\mathrm{T}}$, *y*, and $L_{xyz}$) for the $\mathrm{p}\mathrm{p}$ results. After checking that the efficiencies on the prompt and nonprompt ${\mathrm{J}}/\psi$ MC samples near $L_{xyz}=0$ are in agreement, two efficiency calculations are made. One calculation is made on the prompt ${\mathrm{J}}/\psi$ MC sample, as a function of $p_{\mathrm{T}}$, in 10 rapidity intervals between $y=-2.4$ and $y=2.4$, and 4 centrality bins (0–10%, 10–20%, 20–40%, and 40–100%). For each *y* and centrality interval, the $p_{\mathrm{T}}$ dependence of the efficiency is smoothed by fitting it with a Gaussian error function. A second efficiency is calculated using the nonprompt ${\mathrm{J}}/\psi$ MC sample, as a function of $L_{xyz}$, in the same *y* binning, but for coarser $p_{\mathrm{T}}$ bins and for centrality 0–100%. This is done in two steps. The efficiency is first calculated as a function of $L_{xyz}^{\text {true}}$, and then converted into an efficiency versus measured $L_{xyz}$, using a 2D dispersion map of $L_{xyz}^{\text {true}}$ *vs.* $L_{xyz}$. In the end, each dimuon candidate selected in data, with transverse momentum $p_{\mathrm{T}}$, rapidity *y*, centrality *c*, and $L_{xyz}=d$ (mm), is assigned an efficiency weight equal to

1 $$\begin{aligned} w= & {} \text {efficiency}^{\text {prompt} {\mathrm{J}}/\psi }(p_{\mathrm{T}},y,c,L_{xyz}=0)\nonumber \\&\times \frac{\text {efficiency}^{\text {nonprompt}\,{\mathrm{J}}/\psi }(p_{\mathrm{T}},y,L_{xyz}=d)}{\text {efficiency}^{\text {nonprompt}\,{\mathrm{J}}/\psi }(p_{\mathrm{T}},y,L_{xyz}=0)}. \end{aligned}$$

The individual components of the MC efficiency (tracking reconstruction, standalone muon reconstruction, global muon fit, muon identification and selection, triggering) are cross-checked using single muons from ${\mathrm{J}}/\psi$ decays in simulated and collision data, with the *tag-and-probe* technique (T&P)  [53]. For all but the tracking reconstruction, scaling factors (calculated as the ratios between the data and MC T&P obtained efficiencies), estimated as a function of the muon $p_{\mathrm{T}}$ in several muon pseudorapidity regions, are used to scale the dimuon MC-calculated efficiencies. They are applied event-by-event, as a weight, to each muon that passes all analysis selections and enter the mass and $\ell _{{\mathrm{J}}/\psi }$ distributions. The weights are similar for the $\mathrm{p}\mathrm{p}$ and $\mathrm{PbPb}$ samples, and range from 1.02 to 0.6 for single muons with $p_{\mathrm{T}} >4-5$ $\,{\mathrm{GeV}}/{\mathrm{c}}$ and $p_{\mathrm{T}} <3.5$ $\,{\mathrm{GeV}}/{\mathrm{c}}$, respectively. For the tracking efficiency, which is above 99% even in the case of PbPb events, the full difference between data and MC T&P results (integrated over all the kinematic region probed) is propagated as a global (common to all points) systematic uncertainty.

### Signal extraction

The single-muon acceptance and identification criteria are the same as in Ref. [22]. Opposite-charge muon pairs, with invariant mass between 2.6 and 3.5${\,\text {GeV/}c^{2}}$, are fitted with a common vertex constraint and are kept if the fit $\chi ^2$ probability is larger than 1%. Results are presented in up to six bins of absolute ${\mathrm{J}}/\psi$ meson rapidity (equally spaced between 0 and 2.4) integrated over $p_{\mathrm{T}}$ (6.5 $<$ $p_{\mathrm{T}}$ $<$ 30 GeV/*c*), up to six bins in $p_{\mathrm{T}}$ ([6.5, 8.5], [8.5, 9.5], [9.5, 11], [11, 13], [13, 16], [16, 30] $\,{\mathrm{GeV}}/{\mathrm{c}}$) integrated over rapidity ($|{y}|<2.4$), and up to three additional low-$p_{\mathrm{T}}$ bins ([3, 4.5], [4.5, 5.5], [5.5, 6.5]$\,{\mathrm{GeV}}/{\mathrm{c}}$) at forward rapidity ($1.6<|{y}|<2.4$). The lower $p_{\mathrm{T}}$ limit for which the results are reported is imposed by the detector acceptance, the muon reconstruction algorithm, and the selection criteria used in the analysis. The $\mathrm{PbPb}$ sample is split in bins of collision centrality, defined using fractions of the inelastic hadronic cross section where 0% denotes the most central collisions. This fraction is determined from the HF energy distribution [54]. The most central (highest HF energy deposit) and most peripheral (lowest HF energy deposit) centrality bins used in the analysis are 0–5% and 60–100%, and 0–10% and 50–100%, for prompt and nonprompt ${\mathrm{J}}/\psi$ results, respectively. The rest of the centrality bins are in increments of 5% up to 50% for the high $p_{\mathrm{T}}$ prompt ${\mathrm{J}}/\psi$ results integrated over *y*, and in increments of 10% for all other cases. The $N_{\text {part}}$ values, computed for events with a flat centrality distribution, range from $381\pm 2$ in the 0–5% bin to $14\pm 2$ in the 60–100% bin. If the events would be distributed according to the number of NN collisions, $N_{\text {coll}}$, which is expected for initially produced hard probes, the average $N_{\text {part}}$ would become 25 instead of 14 for the most peripheral bin, and 41 instead of 22 in the case of the 50–100% bin. For the other finer bins, the difference is negligible (less than 3%).

The same method for signal extraction is used in both the $v_{2}$ and the $R_{\mathrm{AA}}$ analyses, for both the $\mathrm{PbPb}$ and $\mathrm{p}\mathrm{p}$ samples. The separation of prompt ${\mathrm{J}}/\psi$ mesons from those coming from $\mathrm{b}$ hadron decays relies on the measurement of a secondary $\mu ^{+} \mu ^{-}$ vertex displaced from the primary collision vertex. The displacement $\vec {r}$ between the $\mu ^{+} \mu ^{-}$ vertex and the primary vertex is measured first. Then, the most probable decay length of $\mathrm{b}$ hadron in the laboratory frame [55] is calculated as

2 $$\begin{aligned} L_{xyz} = \frac{\hat{u}^TS^{-1}\vec {r}}{\hat{u}^TS^{-1}\hat{u}}, \end{aligned}$$

where $\hat{u}$ is the unit vector in the direction of the ${\mathrm{J}}/\psi$ meson momentum ($\vec {p}$) and *S* is the sum of the primary and secondary vertex covariance matrices. From this quantity, the pseudo-proper decay length $\ell _{{\mathrm{J}}/\psi } = L_{xyz}\, m_{{\mathrm{J}}/\psi }/p$ (which is the decay length of the ${\mathrm{J}}/\psi$ meson) is computed as an estimate of the $\mathrm{b}$ hadron decay length.

To measure the fraction of the ${\mathrm{J}}/\psi$ mesons coming from b hadron decays (the so-called *b fraction*), the invariant-mass spectrum of $\mu ^{+} \mu ^{-}$ pairs and their $\ell _{{\mathrm{J}}/\psi }$ distribution are fitted sequentially in an extended unbinned maximum likelihood fit. The fits are performed for each $p_{\mathrm{T}}$, $|{y}|$, and centrality bin of the analysis, and in addition in the case of the $\mathrm{PbPb}$ $v_{2}$ analysis, in four bins in $|{\Delta \Phi }| = |{\phi -\Psi _2}|$, equally spaced between 0 and $\pi /2$. The second-order “event plane” angle $\Psi _2$, measured as explained below, corresponds to the event-by-event azimuthal angle of maximum particle density. It is an approximation of the participant plane angle $\Psi _{\mathrm{PP}}$, which is not directly observable.

The fitting procedure is similar to the one used in earlier analyses of pp collisions at $\sqrt{s}$ = 7$\,\mathrm{TeV}$  [56], and PbPb collisions at $\sqrt{{s_{_{\text {NN}}}}}$ = 2.76$\,\mathrm{TeV}$  [22]. The ${\mathrm{J}}/\psi$ meson mass distribution is modelled by the sum of a Gaussian function and a Crystal Ball (CB) function [57], with a common mean $m_0$ and independent widths. The CB radiative tail parameters are fixed to the values obtained in fits to simulated distributions for different kinematic regions [50]. The invariant mass background probability density function (PDF) is an exponential function whose parameters are allowed to float in each fit. Since the mass resolution depends on *y* and $p_{\mathrm{T}}$, all resolution-related parameters are left free when binning as a function of $|{y}|$ or $p_{\mathrm{T}}$. In the case of centrality binning, the width of the CB function is left free, while the rest of the parameters are fixed to the centrality-integrated results, 0–100%, for a given $p_{\mathrm{T}}$ and $|{y}|$ bin. When binning in $|{\Delta \Phi }|$, all signal parameters are fixed to their values in the $|{\Delta \Phi }|$-integrated fit.

**Fig. 1:**
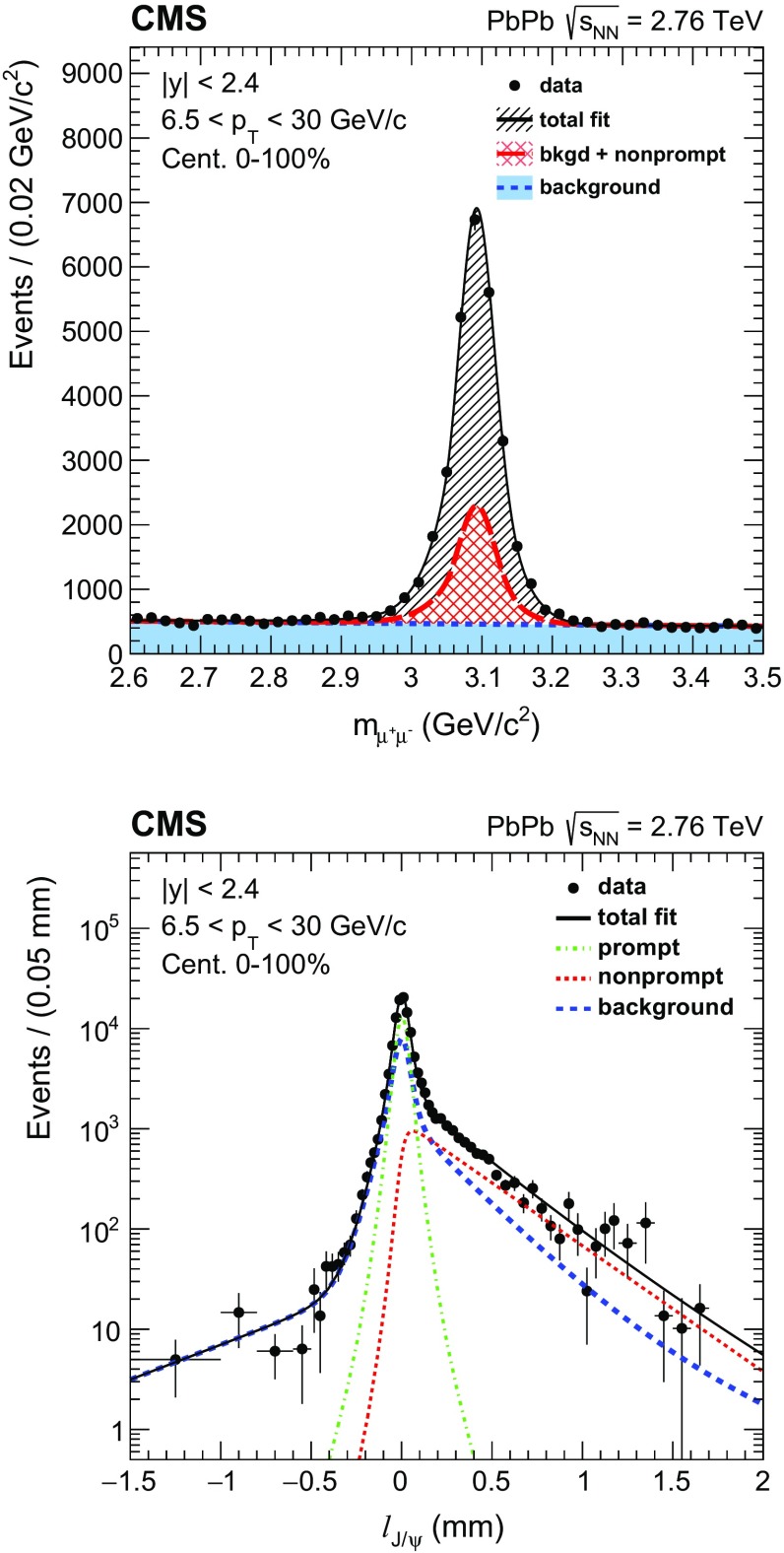
Invariant mass spectra (*top*) and pseudo-proper decay length distribution (*bottom*) of $\mu ^{+} \mu ^{-}$ pairs in centrality 0–100% and integrated over the rapidity range $|{y}|<2.4$ and the $p_{\mathrm{T}}$ range $6.5<p_{\mathrm{T}} <30\,{\mathrm{GeV}}/{\mathrm{c}}$. The *error bars* on each point represent statistical uncertainties. The projections of the two-dimensional fit onto the respective axes are overlaid as *solid black lines*. The *dashed green* and *red lines* show the fitted contribution of prompt and nonprompt ${\mathrm{J}}/\psi$. The fitted background contributions are shown as *dotted blue lines*

The $\ell _{{\mathrm{J}}/\psi }$ distribution is modeled by a prompt signal component represented by a resolution function, a nonprompt component given by an exponential function convoluted with the resolution function, and the continuum background component represented by the sum of the resolution function plus three exponential decay functions to take into account long-lived background components [56]. The resolution function is comprised of the sum of two Gaussian functions, which depend upon the per-event uncertainty of the measured $\ell _{{\mathrm{J}}/\psi }$, determined from the covariance matrices of the primary and secondary vertex fits. The fit parameters of the $\ell _{{\mathrm{J}}/\psi }$ distribution were determined through a series of fits. Pseudo-proper decay length background function parameters are fixed using dimuon events in data located on each side of the ${\mathrm{J}}/\psi$ resonance peak. In all cases, the b fraction is a free fit parameter. An example of 2D fits is given in Fig. 1.

**Fig. 2:**
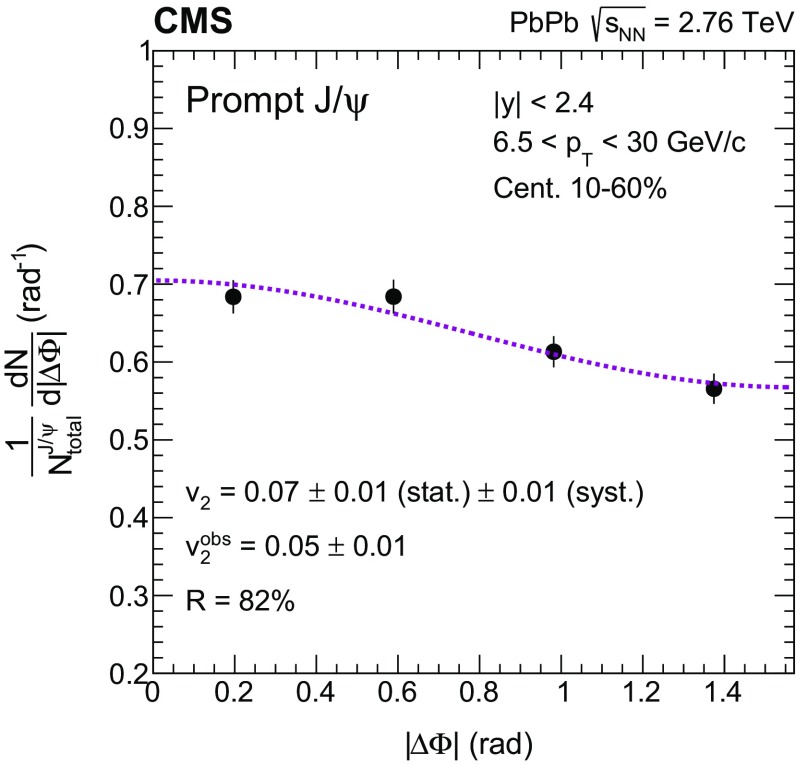
The $|{\Delta \Phi }|$ distribution of high $p_{\mathrm{T}}$ prompt ${\mathrm{J}}/\psi$ mesons, $6.5<p_{\mathrm{T}} <30$ $\,{\mathrm{GeV}}/{\mathrm{c}}$, measured in the rapidity range $|{y}|<2.4$ and event centrality 10–60%, normalized by the bin width and the sum of the prompt yields in all four $\Delta \Phi$ bins. The *dashed line* represents the function $1+2v_{2} ^{\text {obs}} \cos (|{2\Delta \varPhi }|)$ used to extract the $v_{2} ^{\text {obs}}$. The event-averaged resolution correction factor, corresponding to this event centrality, is also listed, together with the calculated final $v_{2}$ for this kinematic bin. The systematic uncertainty listed in the legend includes the 2.7% global uncertainty from the event plane measurement

The $v_{2}$ analysis follows closely the event plane method described in Ref. [58]. The ${\mathrm{J}}/\psi$ mesons reconstructed with $y>0$ ($y<0$) are correlated with the event plane $\Psi _2$ found using energy deposited in a region of the HF spanning $-5< \eta < -3$ ($3< \eta < 5$). This is chosen to introduce a rapidity gap between the particles used in the event plane determination and the ${\mathrm{J}}/\psi$ meson, in order to reduce the effect of other correlations that might exist, such as those from dijet production. To account for nonuniformities in the detector acceptance that can lead to artificial asymmetries in the event plane angle distribution and thereby affect the deduced $v_{2}$ values, a Fourier analysis “flattening” procedure [59] is used, where each calculated event plane angle is shifted slightly to recover a uniform azimuthal distribution, as described in Ref. [58]. The event plane has a resolution that depends on centrality, and is caused by the finite number of particles used in its determination.

The corrections applied event-by-event ensure that the prompt and nonprompt yields extracted from fitting the invariant mass and $\ell _{{\mathrm{J}}/\psi }$ distributions account for reconstruction and selection inefficiencies. As such, after extracting the yields in each $|{y}|$, $p_{\mathrm{T}}$, centrality (and $|{\Delta \Phi }|$) bin, the $v_{2}$ and $R_{\mathrm{AA}}$ can be calculated directly. The $R_{\mathrm{AA}}$ is defined by

3 $$\begin{aligned} R_{\mathrm{AA}} = \frac{N_{\text {PbPb}}^{{\mathrm{J}}/\psi }}{(T_{\mathrm{AA}} \, \sigma _{\text {pp}}^{{\mathrm{J}}/\psi })}, \end{aligned}$$

where $N_{\text {PbPb}}^{{\mathrm{J}}/\psi }$ is the number of prompt or nonprompt ${\mathrm{J}}/\psi$ mesons produced per $\mathrm{PbPb}$ collision, $\sigma _{\text {pp}}^{{\mathrm{J}}/\psi }$ is the corresponding pp cross section, and $T_{\mathrm{AA}}$ is the nuclear overlap function.

The $v_{2}$ is calculated by fitting the $[1/N_{\text {total}}^{{\mathrm{J}}/\psi }][\mathrm{d}N^{{\mathrm{J}}/\psi }/\mathrm{d}|{\Delta \Phi }|]$ distributions with the function $1+2v_{2} ^{\text {obs}} \cos (|{2\Delta \Phi }|)$, where the $N_{\text {total}}^{{\mathrm{J}}/\psi }$ is the prompt or nonprompt ${\mathrm{J}}/\psi$ yield integrated over azimuth for each kinematic bin. An example of such a fit is shown in Fig. 2. The final $v_{2}$ coefficient in the event plane method is evaluated by dividing the observed value $v_2^{\text {obs}}$ by an event-averaged resolution-correction *R*, i.e. $v_2=v_2^{\text {obs}}/R$, as described in Ref. [60]. The factor *R*, calculated experimentally as described in Ref. [58], can range from 0 to 1, with a better resolution corresponding to a larger value of *R*. No difference is observed when determining *R* using the dimuon-triggered events analysed here, compared to the values used in Ref. [58] for the analysis of charged hadrons. For this paper, the $v_{2}$ analysis is restricted to the centrality interval 10–60% to ensure a nonsymmetric overlap region in the colliding nuclei, while maintaining a good event plane resolution ($R \gtrsim 0.8$ in the event centrality ranges in which results are reported: 10–20%, 20–30%, and 30–60%).

### Estimation of uncertainties

Several sources of systematic uncertainties are considered for both $R_{\mathrm{AA}}$ and $v_{2}$ analyses. They are mostly common, thus calculated and propagated in a similar way.

The systematic uncertainties in the signal extraction method (fitting) are evaluated by varying the analytical form of each component of the PDF hypotheses. For the invariant mass PDF, as an alternative signal shape, a sum of two Gaussian functions is used, with shared mean and both widths as free parameters in the fit. For the same PDF, the uncertainty in the background shape is evaluated using a first order Chebychev polynomial. For the differential centrality bins, with the invariant mass signal PDF parameters fixed to the 0–100% bin, an uncertainty is calculated by performing fits in which the constrained parameters are allowed to vary with a Gaussian PDF. The mean of the constraining Gaussian function and the initial value of the constrained parameters come from the fitting in the 0–100% bin with no fixed parameters. The uncertainties of the parameters in the 0–100% bin is used as a width of the constraining Gaussian. For the lifetime PDF components, the settings that could potentially affect the b fraction are changed. The $\ell _{{\mathrm{J}}/\psi }$ shape of the nonprompt ${\mathrm{J}}/\psi$ is taken directly from the reconstructed one in simulation and converted to a PDF. Tails of this PDF, where the MC statistics are insufficient, are mirrored from neighboring points, weighted with the corresponding efficiency. The sum in quadrature of all yield variations with respect to the nominal fit is propagated in the calculation of the systematic uncertainty in the final results. The variations across all $R_{\mathrm{AA}}$ ($v_{2}$) analysis bins are between 0.7 and 16% (2.6 and 38%) for prompt ${\mathrm{J}}/\psi$, and 1.4 and 19% (20 and 81%) for nonprompt ${\mathrm{J}}/\psi$. They increase from mid to forward rapidity, from high- to low-$p_{\mathrm{T}}$, and for $\mathrm{PbPb}$ results also from central to peripheral bins.

Three independent uncertainties are assigned for the dimuon efficiency corrections. One addresses the uncertainty on the parametrization of the efficiency *vs.* $p_{\mathrm{T}}$, *y*, and centrality. For the $R_{\mathrm{AA}}$ results, it is estimated, in each signal *y* and centrality bin, by randomly moving 100 times, each individual efficiency versus $p_{\mathrm{T}}$ point within its statistical uncertainty, re-fitting with the Gaussian error function, and recalculating each time a corrected MC signal yield. For the $v_{2}$ results, this procedure is not practical: it requires re-weighting and re-fitting many times the full data sample. So in this case, the uncertainty is estimated by changing two settings in the nominal efficiency, and re-fitting data only once, with the modified efficiency: (a) using binned efficiency instead of fits, and (b) using only the nonprompt ${\mathrm{J}}/\psi$ MC sample, integrated over all event centralities. The relative uncertainties for this source, propagated into the final results, are calculated for $R_{\mathrm{AA}}$ as the root-mean-square of the 100 yield variations with respect to the yield obtained with the nominal efficiency parametrization, and for the $v_{2}$ analysis as the full difference between the nominal and the modified-efficiency results. Across all $R_{\mathrm{AA}}$ ($v_{2}$) analysis bins, the values are between 0.6 and 20% (1.5 and 54%) for prompt ${\mathrm{J}}/\psi$, and 0.7 and 24% (6.1 and 50%) for nonprompt ${\mathrm{J}}/\psi$ results. These uncertainties increase from high to low $p_{\mathrm{T}}$, and from mid to forward rapidity but do not have a strong centrality dependence.

A second uncertainty addresses the accuracy of the efficiency *vs.* $L_{xyz}$ calculation, and is estimated by changing the $L_{xyz}$ resolution. It is done in several steps: (a) the binning in the $L_{xyz}^{\text {true}}$ *vs.* $L_{xyz}$ maps is changed; (b) the dimuon efficiency weights are recalculated; c) the data is reweighed and refitted to extract the signal yields. The variations across all $R_{\mathrm{AA}}$ ($v_{2}$) analysis bins are between 0.025 and 3.7% (0.1 and 16%) for prompt ${\mathrm{J}}/\psi$, and 0.1 and 13% (29 and 32%) for nonprompt ${\mathrm{J}}/\psi$ results. In the case of the prompt ${\mathrm{J}}/\psi$, the variations are small and rather constant across all bins, around 2-3%, with the 16% variation being reached only in the lowest-$p_{\mathrm{T}}$ bin in the $v_{2}$ analysis. For nonprompt ${\mathrm{J}}/\psi$ the variations increase from mid to forward rapidity, and for $\mathrm{PbPb}$ also from peripheral to central bins.

Finally, a third class of uncertainty arises from the scaling factors. For the $v_{2}$ analysis, the full difference between results with and without T&P corrections is propagated to the final systematic uncertainty. It varies between 0.4 and 7.4% for prompt ${\mathrm{J}}/\psi$, and 5.4 and 8.8% for nonprompt ${\mathrm{J}}/\psi$ results. For the $R_{\mathrm{AA}}$ analysis, this uncertainty comprises two contributions. A parametrization uncertainty was estimated by randomly moving each of the data T&P efficiency points within their statistical uncertainty, recalculating each time the scaling factors and the dimuon efficiencies in all the analysis bins, and propagating the root-mean-square of all variations to the total T&P uncertainty. In addition, a systematic uncertainty was estimated by changing different settings of the T&P method. The contributions are similar for the prompt and nonprompt ${\mathrm{J}}/\psi$ results, and vary between 1.4 and 13% across all bins, for the combined trigger, identification, and reconstruction efficiencies, with the largest uncertainties in the forward and low $p_{\mathrm{T}}$ regions. On top of these bin-by-bin T&P uncertainties, an uncertainty in the tracking reconstruction efficiency, 0.3 and 0.6% for each muon track, for $\mathrm{p}\mathrm{p}$ and $\mathrm{PbPb}$, respectively, is doubled for dimuon candidates, and considered as a global uncertainty in the final results.

There is one additional source of uncertainty that is particular to each analysis. For the $R_{\mathrm{AA}}$ results, it is the $T_{\mathrm{AA}}$ uncertainty, which varies between 16 and 4.1% from most peripheral (70–100%) to most central (0–5%) events, and it has a value of 5.6% for the 0–100% case, estimated as described in Ref. [36]. For the $v_{2}$ analysis, uncertainties are assigned for the event plane measurement. A systematic uncertainty is associated with the event plane flattening procedure and the resolution correction determination (±1% [60]), and another with the sensitivity of the measured $v_{2}$ values to the size of the minimum $\eta$ gap (2.5%, following Ref. [60]). The two uncertainties are added quadratically to a total of 2.7% global uncertainty in the $v_{2}$ measurement.

The total systematic uncertainty in the $R_{\mathrm{AA}}$ is estimated by summing in quadrature the uncertainties from the signal extraction and efficiency weighting. The range of the final uncertainties on prompt and nonprompt ${\mathrm{J}}/\psi$ $R_{\mathrm{AA}}$ is between 2.1 and 22%, and 2.8 and 28%, respectively, across bins of the analysis. The uncertainty in the integrated luminosity of the $\mathrm{p}\mathrm{p}$ data (3.7%), $N_{\mathrm{MB}}$ events in $\mathrm{PbPb}$ data (3%), and tracking efficiency (0.6% for pp and 1.2% for $\mathrm{PbPb}$ data) are considered as global uncertainties.

The total systematic uncertainty for $v_{2}$ is estimated by summing in quadrature the contributions from the yield extraction and efficiency corrections. The range of the final uncertainties on prompt and nonprompt ${\mathrm{J}}/\psi$ $v_{2}$ results is between 10 and 57%, and 37 and 100%, respectively.

### Displaying uncertainties

In all the results shown, statistical uncertainties are represented by error bars, and systematic uncertainties by boxes centered on the points. For the $v_{2}$ results, the global uncertainty from the event plane measurement is not included in the point-by-point uncertainties. Boxes plotted at $R_{\mathrm{AA}} =1$ represent the scale of the global uncertainties. For $R_{\mathrm{AA}}$ results plotted as a function of $p_{\mathrm{T}}$ or $|{y}|$, the statistical and systematic uncertainties include the statistical and systematic components from both $\mathrm{PbPb}$ and $\mathrm{p}\mathrm{p}$ samples, added in quadrature. For these types of results, the systematic uncertainty on $T_{\mathrm{AA}}$, the $\mathrm{p}\mathrm{p}$ sample integrated luminosity uncertainty, the uncertainty in the $N_{\mathrm{MB}}$ of $\mathrm{PbPb}$ events, and the tracking efficiency are added in quadrature and shown as a global uncertainty.

For $R_{\mathrm{AA}}$ results shown as a function of $N_{\text {part}}$, the uncertainties on $T_{\mathrm{AA}}$ are included in the systematic uncertainty, point-by-point. The global uncertainty plotted at $R_{\mathrm{AA}} =1$ as a grey box includes in this case the statistical and systematic uncertainties from the $\mathrm{p}\mathrm{p}$ measurement, the integrated luminosity uncertainty for the $\mathrm{p}\mathrm{p}$ data, the uncertainty in the $N_{\mathrm{MB}}$ of $\mathrm{PbPb}$ events, and the tracking efficiency uncertainty, added in quadrature. When showing $R_{\mathrm{AA}}$ *vs.* $N_{\text {part}}$ separately for different $p_{\mathrm{T}}$ or $|{y}|$ intervals, the statistical and systematic uncertainties from the $\mathrm{p}\mathrm{p}$ measurement are added together in quadrature and plotted as a coloured box at $R_{\mathrm{AA}} =1$. In addition, a second global uncertainty, that is common for all the $p_{\mathrm{T}}$ and $|{y}|$ bins, is calculated as the quadratic sum of the integrated luminosity uncertainty for pp data, the uncertainty in $N_{\mathrm{MB}}$ of $\mathrm{PbPb}$ events, and the tracking efficiency uncertainty, and is plotted as an empty box at $R_{\mathrm{AA}} =1$.

## Results

For all results plotted versus $p_{\mathrm{T}}$ or $|{y}|$, the abscissae of the points correspond to the centre of the respective bin, and the horizontal error bars reflect the width of the bin. When plotted as a function of centrality, the abscissae are average $N_{\text {part}}$ values corresponding to events flatly distributed across centrality. For the $R_{\mathrm{AA}}$ results, the numerical values of the numerator and denominator of Eq. (3) are available in tabulated form in Appendix A.

### Prompt ${\mathrm{J}}/\psi$

**Fig. 3:**
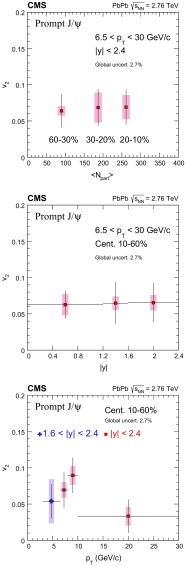
Prompt ${\mathrm{J}}/\psi$ $v_{2}$ as a function of centrality (*top*), rapidity (*middle*), and $p_{\mathrm{T}}$ (*bottom*). The *bars* (*boxes*) represent statistical (systematic) point-by-point uncertainties. The global uncertainty, listed in the legend, is not included in the point-by-point uncertainties. *Horizontal bars* indicate the bin width. The average $N_{\text {part}}$ values correspond to events flatly distributed across centrality

The measured prompt ${\mathrm{J}}/\psi$ $v_{2}$, for 10–60% event centrality and integrated over $6.5<p_{\mathrm{T}} <30$ $\,{\mathrm{GeV}}/{\mathrm{c}}$ and $|{y}|<2.4$, is $0.066 \pm 0.014\,\text {(stat)} \pm 0.014\,\text {(syst)} \pm 0.002\,(\text {global})$. The significance corresponding to a deviation from a $v_{2} =0$ value is 3.3 sigma. Figure 3 shows the dependence of $v_{2}$ on centrality, $|{y}|$, and $p_{\mathrm{T}}$. For each of these results, the dependence on one variable is studied by integrating over the other two. A nonzero $v_{2}$ value is measured in all the kinematic bins studied. The observed anisotropy shows no strong centrality, rapidity, or $p_{\mathrm{T}}$ dependence.

**Fig. 4:**
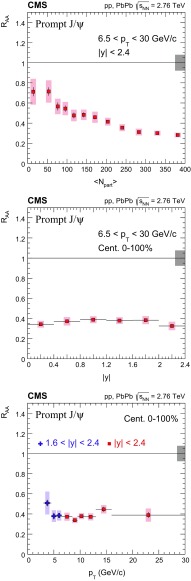
Prompt ${\mathrm{J}}/\psi$ $R_{\mathrm{AA}}$ as a function of centrality (*top*), rapidity (*middle*), and $p_{\mathrm{T}}$ (*bottom*). The *bars* (*boxes*) represent statistical (systematic) point-by-point uncertainties. The *gray boxes* plotted on the right side at $R_{\mathrm{AA}} =1$ represent the scale of the global uncertainties. The average $N_{\text {part}}$ values correspond to events flatly distributed across centrality

In Fig. 4, the $R_{\mathrm{AA}}$ of prompt ${\mathrm{J}}/\psi$ as a function of centrality, $|{y}|$, and $p_{\mathrm{T}}$ are shown, integrating in each case over the other two variables. The $R_{\mathrm{AA}}$ is suppressed even for the most peripheral bin (60–100%), with the suppression slowly increasing with $N_{\text {part}}$. The $R_{\mathrm{AA}}$ for the most central events (0–5%) is measured for $6.5<p_{\mathrm{T}} <30$ $\,{\mathrm{GeV}}/{\mathrm{c}}$ and $|{y}|<2.4$ to be $0.282 \pm 0.010\,\text {(stat)} \pm 0.023\,\text {(syst)}$. No strong rapidity or $p_{\mathrm{T}}$ dependence of the suppression is observed.

**Fig. 5:**
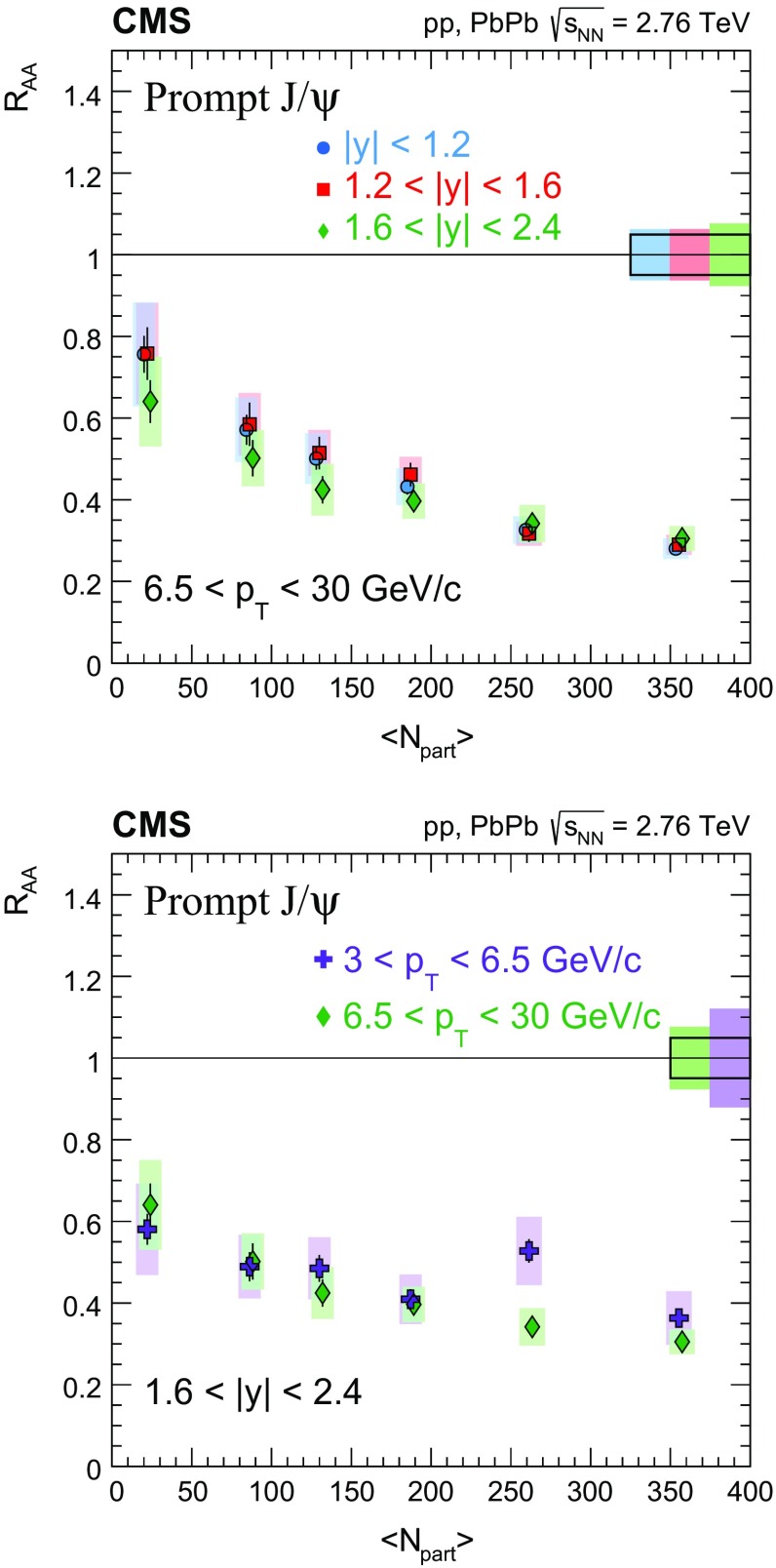
*Top* Prompt ${\mathrm{J}}/\psi$ $R_{\mathrm{AA}}$ as a function of centrality at high $p_{\mathrm{T}}$, $6.5<p_{\mathrm{T}} <30$ $\,{\mathrm{GeV}}/{\mathrm{c}}$, for three different $|{y}|$ regions. The high-$p_{\mathrm{T}}$ mid- and forward-rapidity points are shifted horizontally by $\Delta N_{\text {part}} =2$ for better visibility. *Bottom* Prompt ${\mathrm{J}}/\psi$ $R_{\mathrm{AA}}$ as a function of centrality, at forward rapidity, $1.6<|{y}|<2.4$, for two different $p_{\mathrm{T}}$ regions. The *bars* (*boxes*) represent statistical (systematic) point-by-point uncertainties. The *boxes* plotted on the right side at $R_{\mathrm{AA}} =1$ represent the scale of the global uncertainties: the *coloured boxes* show the statistical and systematic uncertainties from $\mathrm{p}\mathrm{p}$ measurement, and the *open box* shows the global uncertainties common to all data points. The average $N_{\text {part}}$ values correspond to events flatly distributed across centrality

Two double-differential studies are also made, in which a simultaneous binning in centrality and $|{y}|$, or in centrality and $p_{\mathrm{T}}$ is done. Figure 5 (*top*) shows the centrality dependence of high $p_{\mathrm{T}}$ ($6.5<p_{\mathrm{T}} <30$ $\,{\mathrm{GeV}}/{\mathrm{c}}$) prompt ${\mathrm{J}}/\psi$ $R_{\mathrm{AA}}$ measured in three $|{y}|$ intervals. A similar suppression pattern is observed for all rapidities. Figure 5 (*bottom*) shows, for $1.6<|{y}|<2.4$, the $p_{\mathrm{T}}$ dependence of $R_{\mathrm{AA}}$ *vs.* $N_{\text {part}}$. The suppression at low $p_{\mathrm{T}}$ ($3<p_{\mathrm{T}} <6.5$ $\,{\mathrm{GeV}}/{\mathrm{c}}$) is consistent with that at high $p_{\mathrm{T}}$ ($6.5<p_{\mathrm{T}} <30$ $\,{\mathrm{GeV}}/{\mathrm{c}}$).

### Nonprompt ${\mathrm{J}}/\psi$

**Fig. 6:**
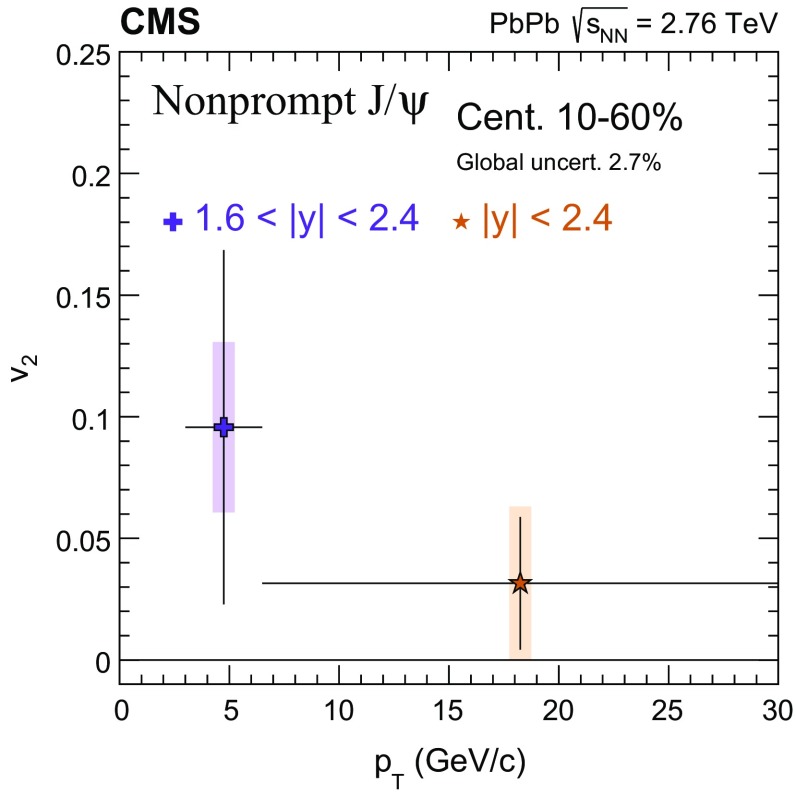
Nonprompt ${\mathrm{J}}/\psi$ $v_{2}$ as a function of $p_{\mathrm{T}}$. The *bars* (*boxes*) represent statistical (systematic) point-by-point uncertainties. The global uncertainty, listed in the legend, is not included in the point-by-point uncertainties. *Horizontal bars* indicate the bin width

Figure 6 shows the nonprompt ${\mathrm{J}}/\psi$ $v_{2}$ *vs.* $p_{\mathrm{T}}$ for 10–60% event centrality, in two kinematic regions: $6.5<p_{\mathrm{T}} <30$ $\,{\mathrm{GeV}}/{\mathrm{c}}$ and $|{y}|<2.4$, and $3<p_{\mathrm{T}} <6.5$ $\,{\mathrm{GeV}}/{\mathrm{c}}$ and $1.6<|{y}|<2.4$. The measured $v_{2}$ for the high-(low-) $p_{\mathrm{T}}$ is $0.032 \pm 0.027 \,\text {(stat)} \pm 0.032 \,\text {(syst)} \pm 0.001 \,(\text {global})$ ($0.096 \pm 0.073 \,\text {(stat)} \pm 0.035 \,\text {(syst)} \pm 0.003 \,(\text {global})$). This is obtained from the fit to the $|\Delta \Phi |$ distribution (as described in Sect. 3.2) with a $\chi ^2$ probability of 22(20)%. Fitting the same distribution with a constant (corresponding to the $v_{2} =0$ case) the $\chi ^2$ probability is 11(8)%. Both measurements are consistent with each other and with a $v_{2}$ value of zero, though both nominal values are positive.

**Fig. 7:**
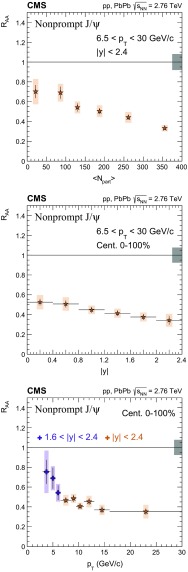
Nonprompt ${\mathrm{J}}/\psi$ $R_{\mathrm{AA}}$ as a function of centrality (*top*), rapidity (*middle*), and $p_{\mathrm{T}}$ (*bottom*). The *bars* (*boxes*) represent statistical (systematic) point-by-point uncertainties. The *gray boxes* plotted on the right side at $R_{\mathrm{AA}} =1$ represent the scale of the global uncertainties. For $R_{\mathrm{AA}}$ *vs.* $N_{\text {part}}$, the average $N_{\text {part}}$ values correspond to events flatly distributed across centrality

In Fig. 7, the $R_{\mathrm{AA}}$ of nonprompt ${\mathrm{J}}/\psi$ as a function of centrality, $|{y}|$, and $p_{\mathrm{T}}$ are shown, integrating in each case over the other two variables. A steady increase of the suppression is observed with increasing centrality of the collision. The $R_{\mathrm{AA}}$ for the most central events (0–10%) measured for $6.5<p_{\mathrm{T}} <30$ $\,{\mathrm{GeV}}/{\mathrm{c}}$ and $|{y}|<2.4$ is $0.332\pm 0.017\,\text {(stat)} \pm 0.028\,\text {(syst)}$. Stronger suppression is observed with both increasing rapidity and $p_{\mathrm{T}}$.

**Fig. 8:**
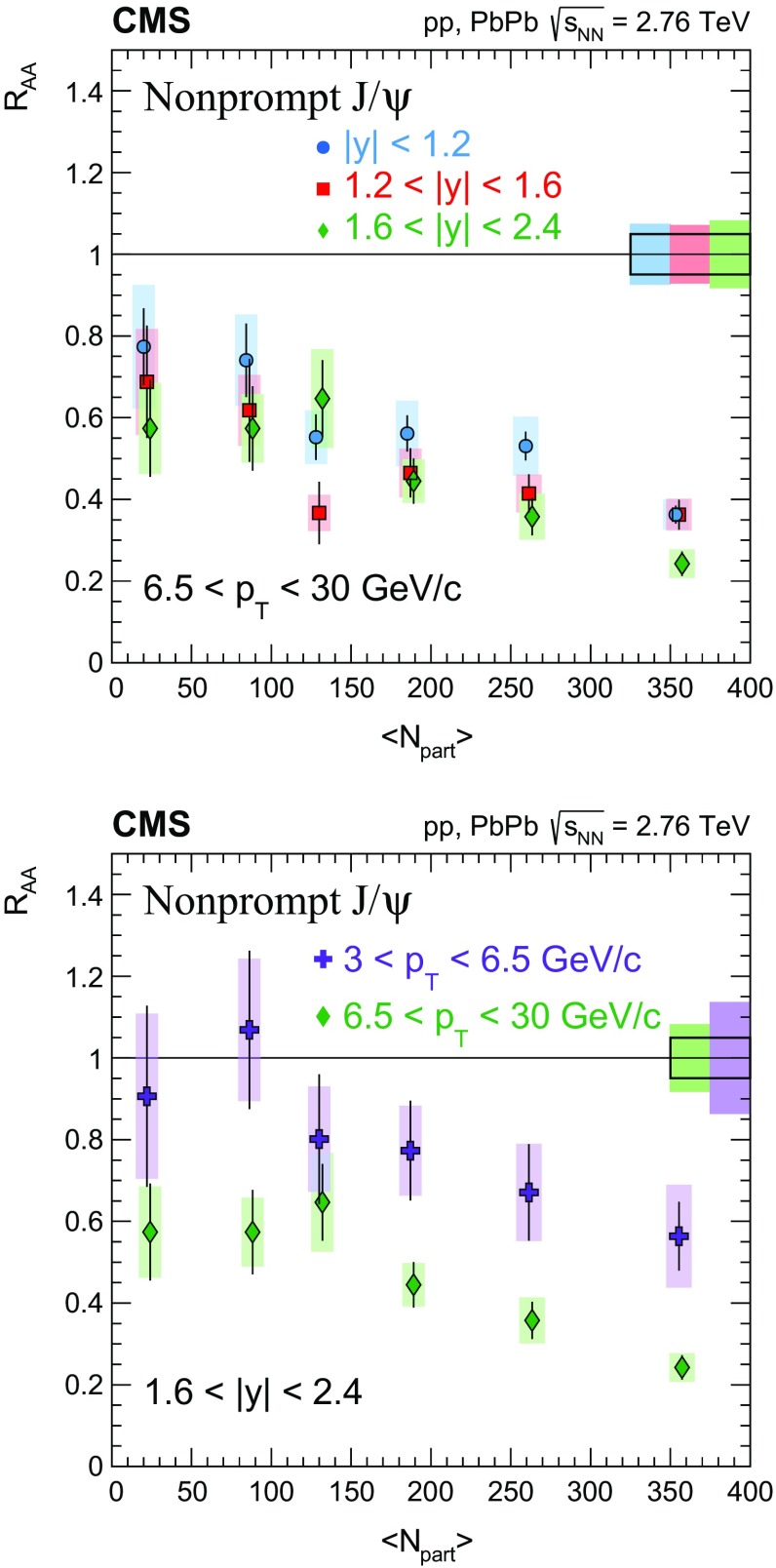
*Top* Nonprompt ${\mathrm{J}}/\psi$ $R_{\mathrm{AA}}$ as a function of centrality at high $p_{\mathrm{T}}$, $6.5<p_{\mathrm{T}} <30$ $\,{\mathrm{GeV}}/{\mathrm{c}}$, for three different $|{y}|$ regions. The high-$p_{\mathrm{T}}$ mid- and forward-rapidity points are shifted horizontally by $\Delta N_{\text {part}} =2$ for better visibility. *Bottom* Nonprompt ${\mathrm{J}}/\psi$ $R_{\mathrm{AA}}$ as a function of centrality, at forward rapidity, $1.6<|{y}|<2.4$, for two different $p_{\mathrm{T}}$ regions. The *bars* (*boxes*) represent statistical (systematic) point-by-point uncertainties. The *boxes* plotted on the right side at $R_{\mathrm{AA}} =1$ represent the scale of the global uncertainties: the *coloured boxes* show the statistical and systematic uncertainties from $\mathrm{p}\mathrm{p}$ measurement, and the *open box* shows the global uncertainties common to all data points. The average $N_{\text {part}}$ values correspond to events flatly distributed across centrality

As for the prompt production case, two double-differential studies were done, simultaneously binning in centrality and $|{y}|$ or $p_{\mathrm{T}}$. Figure 8 (*top*) shows the rapidity dependence of $R_{\mathrm{AA}}$ *vs.* $N_{\text {part}}$ for high $p_{\mathrm{T}}$ nonprompt ${\mathrm{J}}/\psi$. Figure 8 (*bottom*) shows, for $1.6<|{y}|<2.4$, the $p_{\mathrm{T}}$ dependence of $R_{\mathrm{AA}}$ *vs.* $N_{\text {part}}$. The centrality dependences of the three $|{y}|$ intervals are quite similar, and the same is true for the two $p_{\mathrm{T}}$ ranges. As was also seen in Fig. 7, smaller suppression is observed at lower $|{y}|$ and lower $p_{\mathrm{T}}$.

## Discussion

In this section, the $R_{\mathrm{AA}}$ and $v_{2}$ results are compared first for open and hidden charm, and then for open charm and beauty, using data from the ALICE experiment [31, 61, 62]. For open charm, the measurements of $R_{\mathrm{AA}}$ *vs.* $N_{\text {part}}$ of prompt D$^{0}$ mesons, and of averaged prompt $\mathrm{D}$ mesons (${\mathrm{D}^0}$, ${\mathrm{D}^+}$ and $\mathrm{D}^{*+}$ combined), measured in $|{y}|<0.5$ at low $p_{\mathrm{T}}$ ($2<p_{\mathrm{T}} <5$ $\,{\mathrm{GeV}}/{\mathrm{c}}$), and high $p_{\mathrm{T}}$ ($6<p_{\mathrm{T}} <12$ $\,{\mathrm{GeV}}/{\mathrm{c}}$) [61] are used. These are compared to hidden charm data from the prompt ${\mathrm{J}}/\psi$ results described in this paper, in two $p_{\mathrm{T}}$ regions that are similar to the D measurement, i.e. ($3<p_{\mathrm{T}} <6.5$ $\,{\mathrm{GeV}}/{\mathrm{c}}$, $1.6<|{y}|<2.4$) and ($6.5<p_{\mathrm{T}} <30$ $\,{\mathrm{GeV}}/{\mathrm{c}}$, $|{y}|<1.2$). For the $R_{\mathrm{AA}}$ comparison of open charm *vs.* beauty, the averaged prompt D mesons measured in $|{y}|<0.5$ [62] are compared to the nonprompt ${\mathrm{J}}/\psi$ results reported in this paper for $|{y}|<1.2$. The $p_{\mathrm{T}}$ interval ($8<p_{\mathrm{T}} <16$ $\,{\mathrm{GeV}}/{\mathrm{c}}$) for the D is chosen to correspond to that of the parent B mesons of the CMS nonprompt ${\mathrm{J}}/\psi$ result [62].

For the $v_{2}$ results, the $p_{\mathrm{T}}$ dependence reported in this paper for both prompt and nonprompt ${\mathrm{J}}/\psi$ in the centrality 10–60% bin are compared with the $v_{2}$ of the averaged D mesons [31] measured in the 30–50% centrality bin. In addition, the CMS charged-hadron $v_{2}$ results, measured for $|{\eta }|<0.5$, derived for 10–60% centrality bin from Refs. [60] and [58], are added to the comparison.

**Fig. 9:**
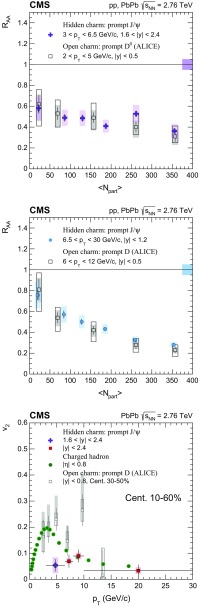
Prompt ${\mathrm{J}}/\psi$ and D meson [61] $R_{\mathrm{AA}}$ *vs.* centrality for low $p_{\mathrm{T}}$ (*top*) and high $p_{\mathrm{T}}$ (*middle*). The average $N_{\text {part}}$ values correspond to events flatly distributed across centrality. *Bottom* Prompt ${\mathrm{J}}/\psi$ and D meson [31], and charged hadron [58, 60] $v_{2}$ *vs.* $p_{\mathrm{T}}$

### Open versus hidden charm

The top two panels of Fig. 9 show the $R_{\mathrm{AA}}$ dependence on the centrality of the prompt ${\mathrm{J}}/\psi$ (bound $\mathrm{Q} \overline{\mathrm{Q}}$ state) and of prompt D (charm-light states $\mathrm{Q} \overline{\mathrm{q}}$) mesons, for low- (*top*) and high- (*middle*) $p_{\mathrm{T}}$ selections. In both cases, the mesons suffer a similar suppression, over the whole $N_{\text {part}}$ range, even though the charmonium yield should be affected by colour screening [4, 48], potentially by final-state nuclear interactions unrelated to the QGP [63–67], and by rather large feed-down contributions from excited states [68, 69]. Moreover, common processes (i.e. recombination or energy loss effects) are expected to affect differently the open and hidden charm [26, 27, 70, 71]. While the present results cannot resolve all these effects, the comparison of open and hidden charm could help to determine their admixture.

A comparison of the $p_{\mathrm{T}}$ dependence of the azimuthal anisotropy $v_{2}$ between the prompt ${\mathrm{J}}/\psi$ and D mesons is made in the bottom panel of Fig. 9. While the $R_{\mathrm{AA}}$ is similar both at low and high $p_{\mathrm{T}}$, the $v_{2}$ of prompt ${\mathrm{J}}/\psi$ at low $p_{\mathrm{T}}$ is lower than that of both D mesons and charged hadrons. At high $p_{\mathrm{T}}$, all three results, within the uncertainties, are similar: the prompt ${\mathrm{J}}/\psi$ results seem to point to a similar anisotropy as the light-quarks hadrons, hinting at a flavour independence of the energy-loss path-length dependence. The prompt ${\mathrm{J}}/\psi$ results could help advance the theoretical knowledge on the relative contribution of the regenerated charmonium yield, as this is the only type of ${\mathrm{J}}/\psi$ expected to be affected by the collective expansion of the medium. Such prompt ${\mathrm{J}}/\psi$ should have higher $v_{2}$ values, closer to those of light-quark hadrons [27].

### Open charm versus beauty

**Fig. 10:**
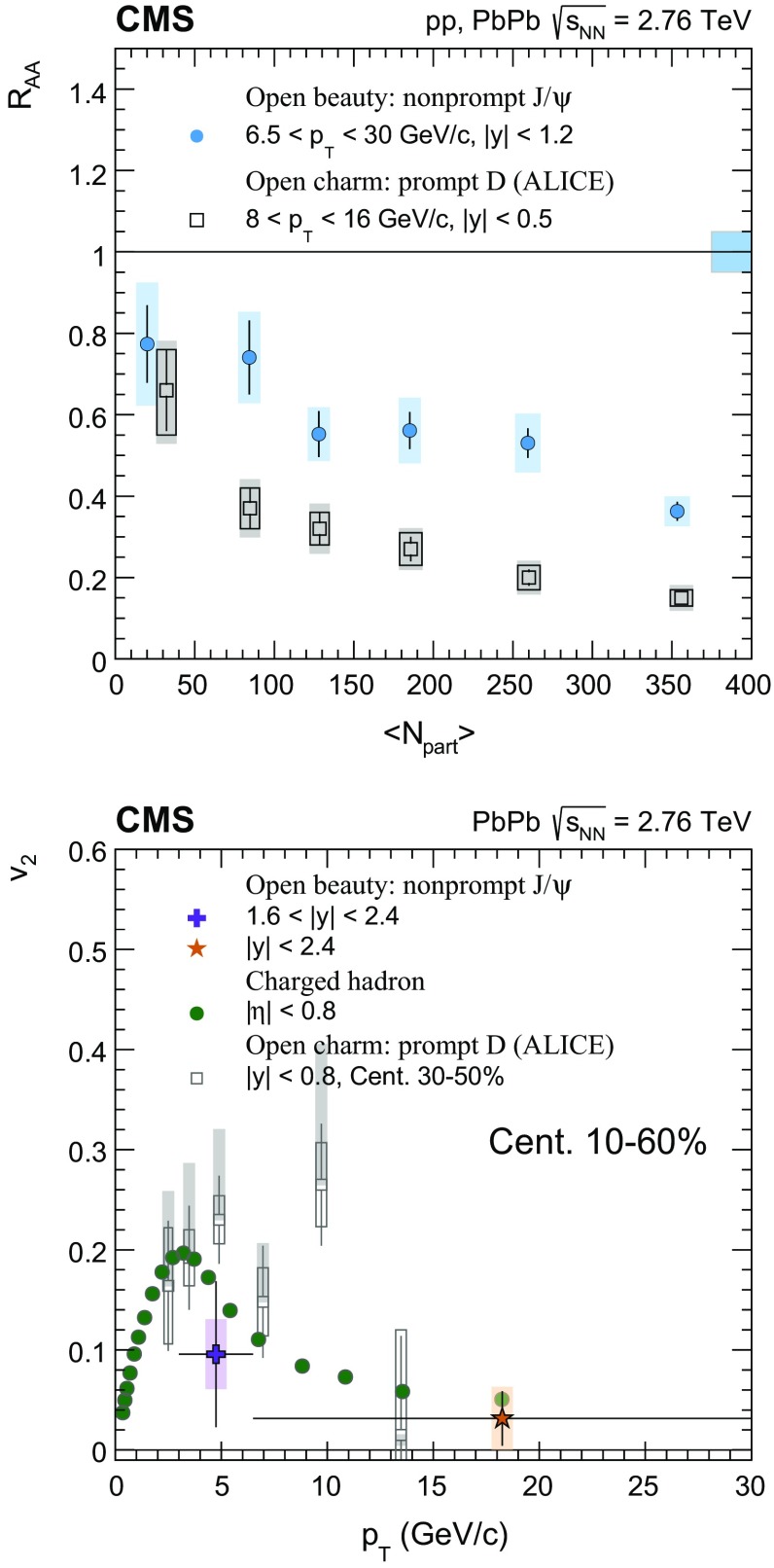
Nonprompt ${\mathrm{J}}/\psi$ and prompt D meson [31, 62], and charged hadron [58, 60] $R_{\mathrm{AA}}$ *vs.* centrality (*top*), and $v_{2}$ *vs.* $p_{\mathrm{T}}$ (*bottom*). For the *top* plot, the average $N_{\text {part}}$ values correspond to events flatly distributed across centrality

The *top* panel of Fig. 10 shows the $R_{\mathrm{AA}}$ dependence on centrality of the nonprompt ${\mathrm{J}}/\psi$ (decay product of B mesons originating from b quarks) and for D mesons (originating from c quarks). The D mesons are more suppressed than the nonprompt ${\mathrm{J}}/\psi$. This is expected in models that assume less radiative energy loss for the b quark compared to that of a c quark because of the ‘dead-cone effect’ (the suppression of gluon bremsstrahlung of a quark with mass *m* and energy *E*, for angles $\theta < m/E$ [72, 73]), and smaller collisional energy loss for the much heavier b quark than for the c quark [15, 74]. The results bring extra information in a kinematic phase space not accessible with fully reconstructed b jet measurements, which show that for $p_{\mathrm{T}} >80$ $\,{\mathrm{GeV}}/{\mathrm{c}}$ the $R_{\mathrm{AA}}$ of b jets is compatible to that of light-quark or gluon jets [75]. However, assessing and quantifying the parton mass dependence of the in-medium phenomena is not trivial: one has to account among other things for different starting kinematics (different unmodified vacuum spectra of the beauty and charm quarks in the medium), and the effect of different fragmentation functions (and extra decay kinematics) [76]. Also, when considering the parton mass dependence, it should be noted that at high-$p_{\mathrm{T}}$, the $R_{\mathrm{AA}}$ of D mesons was found to be similar to that of charged pions over a wide range of event centrality [31].

The *bottom* panel of Fig. 10 shows the $p_{\mathrm{T}}$ dependence of the measured $v_{2}$ for nonprompt ${\mathrm{J}}/\psi$, prompt D mesons, and charged hadrons. The precision and statistical reach of the present LHC open beauty and charm $v_{2}$ results can not answer: (a) at low $p_{\mathrm{T}}$, whether the b quarks, with their mass much larger than that of the charm quarks, participate or not in the collective expansion of the medium as the charm quarks seem to do; (b) at high $p_{\mathrm{T}}$, whether there is a difference in path-length dependence of energy loss between b and c quarks.

## Summary

The production of prompt and nonprompt (coming from b hadron decay) ${\mathrm{J}}/\psi$ has been studied in pp and PbPb collisions at $\sqrt{{s_{_{\text {NN}}}}} = 2.76$ $\,\mathrm{TeV}$. The $R_{\mathrm{AA}}$ of the prompt ${\mathrm{J}}/\psi$ mesons, integrated over the rapidity range $|{y}|<2.4$ and high $p_{\mathrm{T}}$, $6.5<p_{\mathrm{T}} <30$ $\,{\mathrm{GeV}}/{\mathrm{c}}$, is measured in 12 centrality bins. The $R_{\mathrm{AA}}$ is less than unity even in the most peripheral bin, and the suppression becomes steadily stronger as centrality increases. Integrated over rapidity ($p_{\mathrm{T}}$) and centrality, no strong evidence for a $p_{\mathrm{T}}$ (rapidity) dependence of the suppression is found. The azimuthal anisotropy of prompt ${\mathrm{J}}/\psi$ mesons shows a nonzero $v_{2}$ value in all studied bins, while no strong dependence on centrality, rapidity, or $p_{\mathrm{T}}$ is observed.

The $R_{\mathrm{AA}}$ of nonprompt ${\mathrm{J}}/\psi$ mesons shows a slow decrease with increasing centrality and rapidity. The results show less suppression at low $p_{\mathrm{T}}$. The first measurement of the nonprompt ${\mathrm{J}}/\psi$ $v_{2}$ is also reported in two $p_{\mathrm{T}}$ bins for 10–60% event centrality, and the values are consistent with zero elliptical azimuthal anisotropy, though both nominal values are positive.

## Supplemental Material

The nominator and denominator of the $R_{\mathrm{AA}}$, defined in Eq. (3), and presented in this paper in Figs. 4 and 5 for prompt ${\mathrm{J}}/\psi$, and Figs. 7 and 8 for nonprompt ${\mathrm{J}}/\psi$, are tabulated. They represent the efficiency-corrected signal yield within the single muon kinematic region used in this paper. This kinematic region is defined in Eq. (4). These $\sqrt{{s_{_{\text {NN}}}}}$ $=$ 2.76$\,\mathrm{TeV}$ pp and PbPb fiducial cross sections do not depend on the acceptance, or the associated uncertainties. The corresponding $T_{\mathrm{AA}}$ values used in each case are also tabulated.

4 $$\begin{aligned}&p_{\mathrm{T}} ^{\mu }> 3.4\,{\mathrm{GeV}}/{\mathrm{c}} \qquad \qquad \quad \qquad \text { for } |\eta ^{\mu }|< 1.0, \nonumber \\&p_{\mathrm{T}} ^{\mu }> (5.8 - 2.4\,|\eta ^{\mu }|)\,{\mathrm{GeV}}/{\mathrm{c}} \quad \text { for } 1.0< |\eta ^{\mu }|< 1.5, \nonumber \\&p_{\mathrm{T}} ^{\mu } > (3.4 - 0.78\,|\eta ^{\mu }|)\,{\mathrm{GeV}}/{\mathrm{c}} \quad \text { for } 1.5< |\eta ^{\mu }| < 2.4. \end{aligned}$$

### Prompt ${\mathrm{J}}/\psi$

See Tables 1, 2, 3 and 4.

**Table 1:** The prompt ${\mathrm{J}}/\psi$ fiducial cross section in bins of centrality, measured in $\mathrm{PbPb}$ and $\mathrm{p}\mathrm{p}$ collisions at 2.76$\,\mathrm{TeV}$ within the muon acceptance defined by Eq. (4), and the nuclear overlap function ($T_{\mathrm{AA}}$, with its systematic uncertainty). Listed uncertainties are statistical first and systematic second. A global systematic uncertainty of 3.2% (3.7%) affects all $\mathrm{PbPb}$ ($\mathrm{p}\mathrm{p}$) fiducial cross sections. The table corresponds to the top panel of Fig. 4

Centrality (%)	$T_{\mathrm{AA}}$ ($\,\mathrm{mb}^{-1}$)	PbPb	pp
		$\frac{1}{T_{\mathrm{AA}}} \, \frac{\mathrm{d}^3N_{\text {PbPb}}^{{\mathrm{J}}/\psi }}{\mathrm{d}y \mathrm{d}p_{\mathrm{T}} \mathrm{d}\text {Cent.}}$ (pb/${\mathrm{GeV}}/{\mathrm{c}}$)	$\frac{\mathrm{d}^2\sigma _{\text {pp}}^{{\mathrm{J}}/\psi }}{\mathrm{d}y \mathrm{d}p_{\mathrm{T}}}$ (pb/${\mathrm{GeV}}/{\mathrm{c}}$)
$|{y}|<2.4$, $6.5<p_{\mathrm{T}} <30$ $\,{\mathrm{GeV}}/{\mathrm{c}}$			
60–100	$0.246\pm 0.041$	$50\pm 3 \pm 9$	$69.6 \pm 0.6 \pm 4.1$
50–60	$1.36\pm 0.19$	$50 \pm 3 \pm 8$	
45–50	$2.29\pm 0.26$	$39 \pm 3 \pm 5$	
40–45	$3.20 \pm 0.34$	$38 \pm 2 \pm 5$	
35–40	$4.4 \pm 0.4$	$33 \pm 2 \pm 4$	
30–35	$5.8 \pm 0.5$	$34 \pm 2 \pm 4$	
25–30	$7.7 \pm 0.5$	$32 \pm 1 \pm 4$	
20–25	$9.9 \pm 0.6$	$29 \pm 1 \pm 3$	
15–20	$12.7 \pm 0.7$	25 ± 1 ± 2	
10–15	16.2 ± 0.8	21.7 ± 0.9 ± 2.3	
5–10	20.5 ± 0.9	20.9 ± 0.8 ± 1.7	
0–5	25.9 ± 1.1	19.6 ± 0.7 ± 1.6

**Table 2:** The prompt ${\mathrm{J}}/\psi$ fiducial cross section in bins of absolute rapidity, measured in $\mathrm{PbPb}$ and $\mathrm{p}\mathrm{p}$ collisions at 2.76$\,\mathrm{TeV}$ within the muon acceptance defined by Eq. (4), and the nuclear overlap function ($T_{\mathrm{AA}}$, with its systematic uncertainty). Listed uncertainties are statistical first and systematic second. A global systematic uncertainty of 6.5% (3.7%) affects all $\mathrm{PbPb}$ ($\mathrm{p}\mathrm{p}$) fiducial cross sections. The table corresponds to the middle panel of Fig. 4

$|{y}|$	$T_{\mathrm{AA}}$ ($\,\mathrm{mb}^{-1}$)	PbPb	pp
		$\frac{1}{T_{\mathrm{AA}}} \, \frac{\mathrm{d}^2N_{\text {PbPb}}^{{\mathrm{J}}/\psi }}{\mathrm{d}y \mathrm{d}p_{\mathrm{T}}}$ (pb/${\mathrm{GeV}}/{\mathrm{c}}$)	$\frac{\mathrm{d}^2\sigma _{\text {pp}}^{{\mathrm{J}}/\psi }}{\mathrm{d}y \mathrm{d}p_{\mathrm{T}}}$ (pb/${\mathrm{GeV}}/{\mathrm{c}}$)
Cent. 0–100%, $6.5<p_{\mathrm{T}} <30$ $\,{\mathrm{GeV}}/{\mathrm{c}}$			
0.0–0.4	5.67 ± 0.32	18.1 ± 0.6 ± 1.4	53 ± 1 ± 3
0.4–0.8		21.1 ± 0.7 ± 1.8	57 ± 1 ± 4
0.8–1.2		28.7 ± 0.9 ± 2.0	74 ± 1 ± 4
1.2–1.6		36 ± 1 ± 2	94 ± 2 ± 6
1.6–2 .0		38 ± 1 ± 3	98 ± 2 ± 7
2.0–2.4		14.4 ± 0.8 ± 1.4	44 ± 1 ± 4

**Table 3:** The prompt ${\mathrm{J}}/\psi$ fiducial cross section in bins of $p_{\mathrm{T}}$, measured in $\mathrm{PbPb}$ and $\mathrm{p}\mathrm{p}$ collisions at 2.76$\,\mathrm{TeV}$ within the muon acceptance defined by Eq. (4), and the nuclear overlap function ($T_{\mathrm{AA}}$, with its systematic uncertainty). Listed uncertainties are statistical first and systematic second. A global systematic uncertainty of 6.5% (3.7%) affects all $\mathrm{PbPb}$ ($\mathrm{p}\mathrm{p}$) fiducial cross sections. The table corresponds to the bottom panel of Fig. 4

$p_{\mathrm{T}}$ ($\,{\mathrm{GeV}}/{\mathrm{c}}$)	$T_{\mathrm{AA}}$ ($\,\mathrm{mb}^{-1}$)	PbPb	pp
		$\frac{1}{T_{\mathrm{AA}}} \, \frac{\mathrm{d}^2N_{\text {PbPb}}^{{\mathrm{J}}/\psi }}{\mathrm{d}y \mathrm{d}p_{\mathrm{T}}}$ (pb/${\mathrm{GeV}}/{\mathrm{c}}$)	$\frac{\mathrm{d}^2\sigma _{\text {pp}}^{{\mathrm{J}}/\psi }}{\mathrm{d}y \mathrm{d}p_{\mathrm{T}}}$ (pb/${\mathrm{GeV}}/{\mathrm{c}}$)
Cent. 0–100%, $1.6<|{y}|<2.4$			
3–4.5	5.67 ± 0.32	272 ± 16 ± 40	534 ± 10 ± 90
4.5–5.5		181 ± 15 ± 23	478 ± 10 ± 41
5.5–6.5		137 ± 7 ± 14	355 ± 8 ± 28
Cent. 0–100%, $|{y}|<2.4$			
6.5–8.5	5.67 ± 0.32	169 ± 4 ± 14	455 ± 5 ± 33
8.5–9.5		85 ± 3 ± 5	252 ± 5 ± 15
9.5–11		55 ± 2 ± 3	147 ± 3 ± 8
11–13		26 ± 1 ± 2	70 ± 2 ± 4
13–16		11.5 ± 0.5 ± 0.9	25.8 ± 0.8 ± 1.2
16–30		1.25 ± 0.08 ± 0.20	3.23 ± 0.14 ± 0.14

**Table 4:** The prompt ${\mathrm{J}}/\psi$ fiducial cross section in bins of centrality, for three $|{y}|$ and two $p_{\mathrm{T}}$ intervals, measured in $\mathrm{PbPb}$ and $\mathrm{p}\mathrm{p}$ collisions at 2.76$\,\mathrm{TeV}$ within the muon acceptance defined by Eq. (4), and the nuclear overlap function ($T_{\mathrm{AA}}$, with its systematic uncertainty). Listed uncertainties are statistical first and systematic second. A global systematic uncertainty of 3.2% (3.7%) affects all $\mathrm{PbPb}$ ($\mathrm{p}\mathrm{p}$) fiducial cross sections. The table corresponds to Fig. 5

Centrality (%)	$T_{\mathrm{AA}}$ ($\,\mathrm{mb}^{-1}$)	PbPb	pp
		$\frac{1}{T_{\mathrm{AA}}} \, \frac{\mathrm{d}^3N_{\text {PbPb}}^{{\mathrm{J}}/\psi }}{\mathrm{d}y \mathrm{d}p_{\mathrm{T}} \mathrm{d}\text {Cent.}}$ (pb/${\mathrm{GeV}}/{\mathrm{c}}$)	$\frac{\mathrm{d}^2\sigma _{\text {pp}}^{{\mathrm{J}}/\psi }}{\mathrm{d}y \mathrm{d}p_{\mathrm{T}}}$ (pb/${\mathrm{GeV}}/{\mathrm{c}}$)
$0<|{y}|<1.2$, $6.5<p_{\mathrm{T}} <30$ $\,{\mathrm{GeV}}/{\mathrm{c}}$			
50–100	0.468 ± 0.070	47 ± 3 ± 8	61.4 ± 0.7 ± 3.7
40–50	2.75 ± 0.30	35 ± 2 ± 5	
30–40	5.1 ± 0.4	31 ± 2 ± 4	
20–30	8.8 ± 0.6	27 ± 1 ± 3	
10–20	14.5 ± 0.8	20.0 ± 0.8 ± 2.1	
0–10	23 ± 1	17.2 ± 0.7 ± 1.6	
$1.2<|{y}|<1.6$, $6.5<p_{\mathrm{T}} <30$ $\,{\mathrm{GeV}}/{\mathrm{c}}$			
50–100	0.468 ± 0.070	71 ± 6 ± 12	94 ± 2 ± 6
40–50	2.75 ± 0.30	55 ± 5 ± 7	
30–40	5.1 ± 0.4	48 ± 4 ± 5	
20–30	8.8 ± 0.6	43 ± 3 ± 4	
10–20	14.5 ± 0.8	30 ± 2 ± 3	
0–10	23 ± 1	27 ± 1 ± 2	
$1.6<|{y}|<2.4$, $6.5<p_{\mathrm{T}} <30$ $\,{\mathrm{GeV}}/{\mathrm{c}}$			
50–100	0.468 ± 0.070	46 ± 4 ± 8	71 ± 1 ± 5
40–50	2.75 ± 0.30	36 ± 3 ± 5	
30–40	5.1 ± 0.4	30 ± 2 ± 5	
20–30	8.8 ± 0.6	28 ± 2 ± 3	
10–20	14.5 ± 0.8	24 ± 1 ± 3	
0–10	23 ± 1	22 ± 1 ± 2	
$1.6<|{y}|<2.4$, $3<p_{\mathrm{T}} <6.5$ $\,{\mathrm{GeV}}/{\mathrm{c}}$			
50–100	0.468 ± 0.070	815 ± 53 ± 158	1397 ± 16 ± 166
40–50	2.75 ± 0.30	685 ± 50 ± 109	
30–40	5.1 ± 0.4	677 ± 46 ± 107	
20–30	8.8 ± 0.6	572 ± 35 ± 85	
10–20	14.5 ± 0.8	737 ± 40 ± 117	
0–10	23 ± 1	508 ± 29 ± 92	

### Nonprompt ${\mathrm{J}}/\psi$

See Tables 5, 6, 7 and 8.

**Table 5:** The nonprompt ${\mathrm{J}}/\psi$ fiducial cross section in bins of centrality, measured in $\mathrm{PbPb}$ and $\mathrm{p}\mathrm{p}$ collisions at 2.76$\,\mathrm{TeV}$ within the muon acceptance defined by Eq. (4), and the nuclear overlap function ($T_{\mathrm{AA}}$, with its systematic uncertainty). Listed uncertainties are statistical first and systematic second. A global systematic uncertainty of 3.2% (3.7%) affects all $\mathrm{PbPb}$ ($\mathrm{p}\mathrm{p}$) fiducial cross sections. The table corresponds to the top panel of Fig. 7

Centrality (%)	$T_{\mathrm{AA}}$ ($\,\mathrm{mb}^{-1}$)	PbPb	pp
		$\frac{1}{T_{\mathrm{AA}}} \, \frac{\mathrm{d}^3N_{\text {PbPb}}^{{\mathrm{J}}/\psi }}{\mathrm{d}y \mathrm{d}p_{\mathrm{T}} \mathrm{d}\text {Cent.}}$ (pb/${\mathrm{GeV}}/{\mathrm{c}}$)	$\frac{\mathrm{d}^2\sigma _{\text {pp}}^{{\mathrm{J}}/\psi }}{\mathrm{d}y \mathrm{d}p_{\mathrm{T}}}$ (pb/${\mathrm{GeV}}/{\mathrm{c}}$)
$|{y}|<2.4$, $6.5<p_{\mathrm{T}} <30$ $\,{\mathrm{GeV}}/{\mathrm{c}}$			
50–100	0.468 ± 0.070	17 ± 2 ± 3	23.57 ± 0.33 ± 1.41
40–50	2.75 ± 0.30	16 ± 1 ± 2	
30–40	5.1 ± 0.4	13 ± 1 ± 1	
20–30	8.8 ± 0.6	11.9 ± 0.7 ± 1.4	
10–20	14.5 ± 0.8	10.4 ± 0.5 ± 1.3	
0–10	23 ± 1	7.8 ± 0.4 ± 0.7

**Table 6:** The nonprompt ${\mathrm{J}}/\psi$ fiducial cross section in bins of absolute rapidity, measured in $\mathrm{PbPb}$ and $\mathrm{p}\mathrm{p}$ collisions at 2.76$\,\mathrm{TeV}$ within the muon acceptance defined by Eq. (4), and the nuclear overlap function ($T_{\mathrm{AA}}$, with its systematic uncertainty). Listed uncertainties are statistical first and systematic second. A global systematic uncertainty of 6.5% (3.7%) affects all $\mathrm{PbPb}$ ($\mathrm{p}\mathrm{p}$) fiducial cross sections. The table corresponds to the middle panel of Fig. 7

$|{y}|$	$T_{\mathrm{AA}}$ ($\,\mathrm{mb}^{-1}$)	PbPb	pp
		$\frac{1}{T_{\mathrm{AA}}} \, \frac{\mathrm{d}^2N_{\text {PbPb}}^{{\mathrm{J}}/\psi }}{\mathrm{d}y \mathrm{d}p_{\mathrm{T}}}$ (pb/${\mathrm{GeV}}/{\mathrm{c}}$)	$\frac{\mathrm{d}^2\sigma _{\text {pp}}^{{\mathrm{J}}/\psi }}{\mathrm{d}y \mathrm{d}p_{\mathrm{T}}}$ (pb/${\mathrm{GeV}}/{\mathrm{c}}$)
Cent. 0–100%, $6.5<p_{\mathrm{T}} <30$ $\,{\mathrm{GeV}}/{\mathrm{c}}$			
0.0–0.4	5.67 ± 0.32	10.5 ± 0.6 ± 1.3	20.0 ± 0.7 ± 1.3
0.4–0.8		12.1 ± 0.7 ± 1.3	23.8 ± 0.8 ± 1.9
0.8–1.2		11.3 ± 0.6 ± 0.9	25.2 ± 0.8 ± 1.4
1.2–1.6		13.1 ± 0.8 ± 1.2	32 ± 1 ± 2
1.6–2.0		10.7 ± 0.8 ± 1.0	29 ± 1 ± 2
2.0–2.4		4.2 ± 0.5 ± 0.7	12.2 ± 0.7 ± 1.2

**Table 7:** The nonprompt ${\mathrm{J}}/\psi$ fiducial cross section in bins of $p_{\mathrm{T}}$, measured in $\mathrm{PbPb}$ and $\mathrm{p}\mathrm{p}$ collisions at 2.76$\,\mathrm{TeV}$ within the muon acceptance defined by Eq. (4), and the nuclear overlap function ($T_{\mathrm{AA}}$, with its systematic uncertainty). Listed uncertainties are statistical first and systematic second. A global systematic uncertainty of 6.5% (3.7%) affects all $\mathrm{PbPb}$ ($\mathrm{p}\mathrm{p}$) fiducial cross sections. The table corresponds to the bottom panel of Fig. 7

$p_{\mathrm{T}}$ ($\,{\mathrm{GeV}}/{\mathrm{c}}$)	$T_{\mathrm{AA}}$ ($\,\mathrm{mb}^{-1}$)	PbPb	pp
		$\frac{1}{T_{\mathrm{AA}}} \, \frac{\mathrm{d}^2N_{\text {PbPb}}^{{\mathrm{J}}/\psi }}{\mathrm{d}y \mathrm{d}p_{\mathrm{T}}}$ (pb/${\mathrm{GeV}}/{\mathrm{c}}$)	$\frac{\mathrm{d}^2\sigma _{\text {pp}}^{{\mathrm{J}}/\psi }}{\mathrm{d}y \mathrm{d}p_{\mathrm{T}}}$ (pb/${\mathrm{GeV}}/{\mathrm{c}}$)
Cent. 0–100%, $1.6<|{y}|<2.4$			
3–4.5	5.67 ± 0.32	46 ± 7 ± 8	61 ± 4 ± 14
4.5–5.5		43 ± 6 ± 6	63 ± 4 ± 6
5.5–6.5		31 ± 4 ± 4	57 ± 3 ± 5
Cent. 0–100%, $|{y}|<2.4$			
6.5–8.5	5.67 ± 0.32	52 ± 3 ± 4	111 ± 3 ± 9
8.5–9.5		39 ± 2 ± 3	80 ± 3 ± 5
9.5–11		22 ± 1 ± 1	55 ± 2 ± 3
11–13		16 ± 1 ± 2	35 ± 1 ± 2
13–16		6.0 ± 0.5 ± 0.8	16.3 ± 0.7 ± 0.8
16–30		1.071 ± 0.082 ± 0.203	3.04 ± 0.13 ± 0.14

**Table 8:** The nonprompt ${\mathrm{J}}/\psi$ fiducial cross section in bins of centrality, for three $|{y}|$ and two $p_{\mathrm{T}}$ intervals, measured in $\mathrm{PbPb}$ and $\mathrm{p}\mathrm{p}$ collisions at 2.76$\,\mathrm{TeV}$ within the muon acceptance defined by Eq. (4), and the nuclear overlap function ($T_{\mathrm{AA}}$, with its systematic uncertainty). Listed uncertainties are statistical first and systematic second. A global systematic uncertainty of 3.2% (3.7%) affects all $\mathrm{PbPb}$ ($\mathrm{p}\mathrm{p}$) fiducial cross sections. The table corresponds to Fig. 8

Centrality (%)	$T_{\mathrm{AA}}$ ($\,\mathrm{mb}^{-1}$)	PbPb	pp
		$\frac{1}{T_{\mathrm{AA}}} \, \frac{\mathrm{d}^3N_{\text {PbPb}}^{{\mathrm{J}}/\psi }}{\mathrm{d}y \mathrm{d}p_{\mathrm{T}} \mathrm{d}\text {Cent.}}$ (pb/${\mathrm{GeV}}/{\mathrm{c}}$ )	$\frac{\mathrm{d}^2\sigma _{\text {pp}}^{{\mathrm{J}}/\psi }}{\mathrm{d}y \mathrm{d}p_{\mathrm{T}}}$ (pb/${\mathrm{GeV}}/{\mathrm{c}}$ )
$0<|{y}|<1.2$, $6.5<p_{\mathrm{T}} <30$ $\,{\mathrm{GeV}}/{\mathrm{c}}$			
50 ± 100	0.468 ± 0.070	18 ± 2 ± 4	23.3 ± 0.4 ± 1.6
40 ± 50	2.75 ± 0.30	17 ± 2 ± 3	
30 ± 40	5.1 ± 0.4	13 ± 1 ± 2	
20 ± 30	8.8 ± 0.6	13 ± 1 ± 2	
10 ± 20	14.5 ± 0.8	12.4 ± 0.8 ± 1.7	
0 ± 10	23 ± 1	8.5 ± 0.5 ± 0.9	
$1.2<|{y}|<1.6$, $6.5<p_{\mathrm{T}} <30$ $\,{\mathrm{GeV}}/{\mathrm{c}}$			
50 ± 100	0.468 ± 0.070	22 ± 4 ± 4	32 ± 1 ± 2
40 ± 50	2.75 ± 0.30	20 ± 4 ± 3	
30 ± 40	5.1 ± 0.4	12 ± 2 ± 1	
20 ± 30	8.8 ± 0.6	15 ± 2 ± 2	
10 ± 20	14.5 ± 0.8	13 ± 1 ± 1	
0 ± 10	23 ± 1	11 ± 1 ± 1	
$1.6<|{y}|<2.4$, $6.5<p_{\mathrm{T}} <30$ $\,{\mathrm{GeV}}/{\mathrm{c}}$			
50 ± 100	0.468 ± 0.070	12 ± 2 ± 2	20.3 ± 0.6 ± 1.5
40 ± 50	2.75 ± 0.30	12 ± 2 ± 2	
30 ± 40	5.1 ± 0.4	13 ± 2 ± 2	
20 ± 30	8.8 ± 0.6	9 ± 1 ± 1	
10 ± 20	14.5 ± 0.8	7.3 ± 0.9 ± 1.1	
0 ± 10	23 ± 1	4.9 ± 0.6 ± 0.7	
$1.6<|{y}|<2.4$, $3<p_{\mathrm{T}} <6.5$ $\,{\mathrm{GeV}}/{\mathrm{c}}$			
50 ± 100	0.468 ± 0.070	163 ± 40 ± 37	179 ± 7 ± 23
40 ± 50	2.75 ± 0.30	192 ± 35 ± 31	
30 ± 40	5.1 ± 0.4	144 ± 29 ± 23	
20 ± 30	8.8 ± 0.6	139 ± 22 ± 20	
10 ± 20	14.5 ± 0.8	120 ± 21 ± 21	
0 ± 10	23 ± 1	101 ± 15 ± 23	
